# Supplementary material for: Unlocking CRISPR-Cas9 editing for widely diverse Dictyostelid species
Source: Mol Syst Biol. 2026 Jan 12;22(4):599–619. doi: 10.1038/s44320-025-00180-8 (PMC13046832; doi:10.1038/s44320-025-00180-8)
Supplement: Supplementary file 1 — Appendix [file 44320_2025_180_MOESM1_ESM.pdf]

Appendix for

Unlocking CRISPR-Cas9 editing for widely diverse *Dictyostelid* species

Mireia Garriga-Canut, Nikki Cannon, Matt Benton, Andrea Zanon, Samuel T. Horsfield, Jacob Scheurich, Kim Remans, John Lees, Alexandre Paix, Jordi van Gestel

Table of contents

**Appendix Figures**	**Page**
**Appendix Figure S1.** Knock-out efficiency for different cell densities and donor oligo qualities.	4
**Appendix Figure S2.** Knock-out and knock-in efficiencies of SpyCas9 proteins.	5
**Appendix Figure S3.** Aligned Sanger sequencing results of knock-out mutants.	7
**Appendix Figure S4.** Quantification of knock-out mutants through flow cytometry.	8
**Appendix Figure S5.** Comparison between *de novo* assembly and RefSeq genome.	9
**Appendix Figure S6**. Knock-out efficiency for different target sites.	10
**Appendix Figure S7.** Effect of SpyCas9 concentration on editing efficiency.	11
**Appendix Figure S8.** Flow cytometry data of knock-in experiments.	12
**Appendix Figure S9.** CRISPR-Cas9 mediated knock-ins.	13
**Appendix Figure S10.** Quantification of secondary knock-out effect.	15
**Appendix Figure S11.** Quantification of sorting accuracy.	16
**Appendix Figure S12.** CRISPR-Cas9 knock-outs targeting *pkaC.*	17
**Appendix Figure S13.** Sorted plates and PCR confirmation of expression reporters.	19
**Appendix Figure S14.** Simultaneous CRISPR-Cas9 editing of two loci.	21
**Appendix Figure S15.** Post-transfection validation of double knock-in mutant.	22
**Appendix Figure S16**. Double knock-out experiment.	24
**Appendix Figure S17**. Simultaneous knock-out and knock-in CRISPR-Cas9 editing.	26
**Appendix Figure S18.** Phylogenetic tree with representative *Dictyostelid* species.	27

**Captions for Appendix Files**	
**Appendix File S1.** Plasmid maps.	28
**Appendix File S2.** Time-lapse of strains with *ecmA* and *act5* expression reporters.	28
**Appendix File S3.** Raw data slug images.	28
**Appendix File S4.** Raw data cell images.	28
**Appendix File S5.** Videos of *Dictyostelid* species (low zoom).	28
**Appendix File S6.** Videos of *Dictyostelid* species (high zoom).	28
**Appendix File S7.** Sanger sequencing data.	28
**Appendix File S8.** Ungated flow cytometry data.	28

**Appendix Tables**	
**Appendix Table S1.** Strain list.	29
**Appendix Table S2.** List of generated knock-out strains.	29
**Appendix Table S3.** Analysis of knock-out strains using Nanopore sequencing	30
**Appendix Table S4.** List of generated knock-in strains.	31
**Appendix Table S5.** List of crRNAs.	32
**Appendix Table S6.** List of oligos.	33

Appendix Text files
Appendix Text S1. Vector sequences. 36
Appendix Text S2. Detailed protocol. 38

References 61

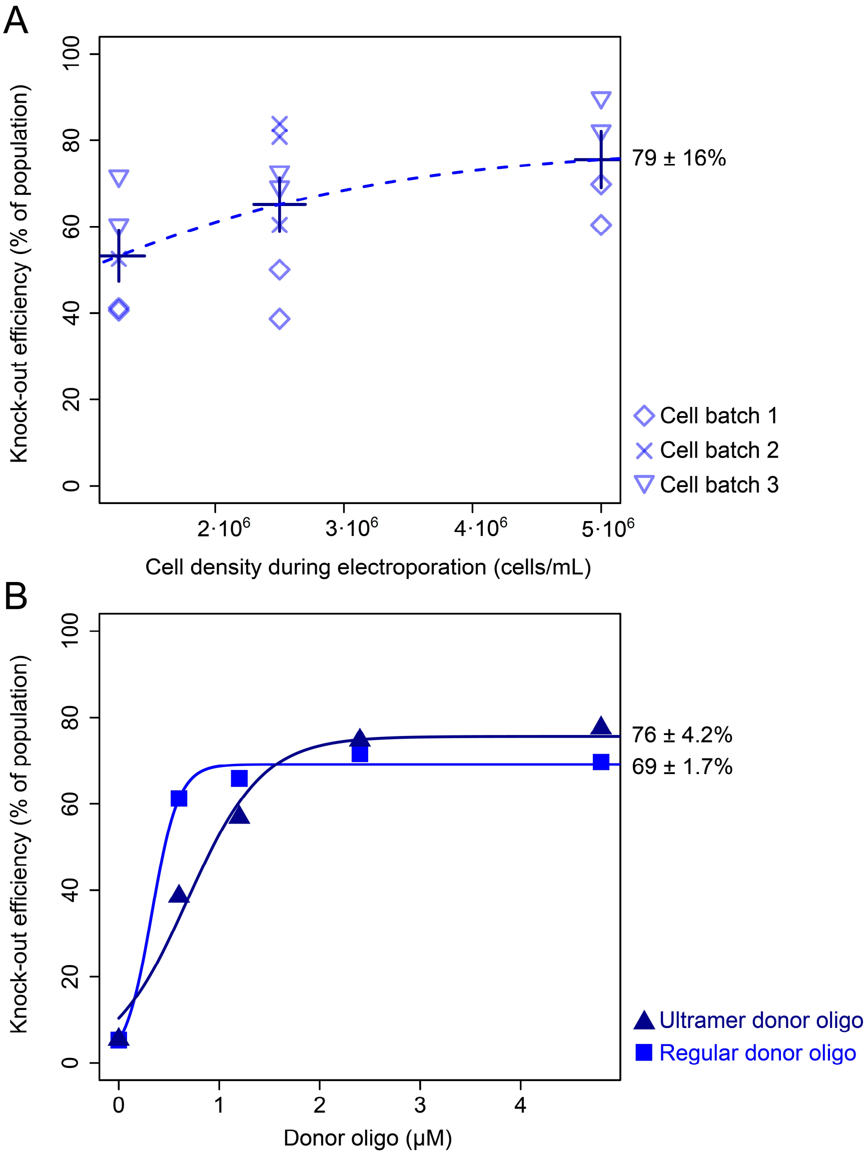

Appendix Figure S1. Knock-out efficiency for different cell densities and donor oligo qualities. (A) Knock-out efficiency using different cell densities per transfection for three batches of axenically-grown *D. discoideum* AX2 *act5*::*mCherry* cells transfected with donor oligo (37bp insertion, 28bp homology arms) targeting aa9 of *mCherry* CDS at 2.4μM, without pre-annealing the gRNA complex. Dashed line indicates non-significant sigmoidal fit. (B) Knock-out efficiency of regular and ultramer oligos produced by IDT. Percentages show estimated maximum knock-out efficiencies and standard errors based on sigmoidal fits. See Appendix Data S1 for data (https://doi.org/10.5281/zenodo.16919775).

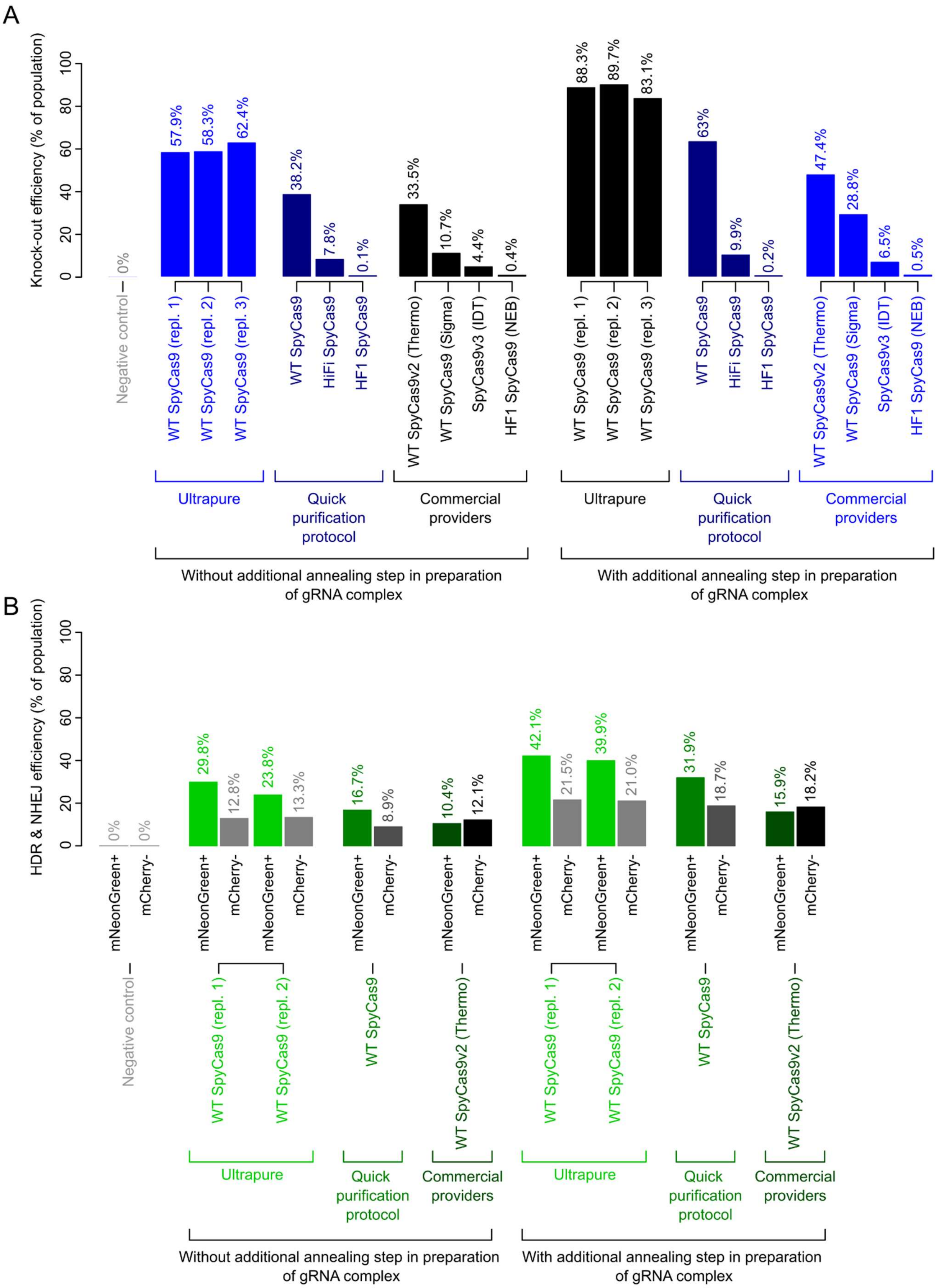

A
Knock-out efficiency (% of population)
0
20
40
60
80
100
0%
57.9%
58.3%
62.4%
38.2%
7.8%
0.1%
33.5%
10.7%
4.4%
0.4%
88.3%
89.7%
83.1%
63%
9.9%
0.2%
47.4%
28.8%
6.5%
0.5%
Negative control
WT SpyCas9 (repl. 1)
WT SpyCas9 (repl. 2)
WT SpyCas9 (repl. 3)
WT SpyCas9
HiFi SpyCas9
HF1 SpyCas9
WT SpyCas9v2 (Thermo)
WT SpyCas9 (Sigma)
SpyCas9v3 (IDT)
HF1 SpyCas9 (NEB)
WT SpyCas9 (repl. 1)
WT SpyCas9 (repl. 2)
WT SpyCas9 (repl. 3)
WT SpyCas9
HiFi SpyCas9
HF1 SpyCas9
WT SpyCas9v2 (Thermo)
WT SpyCas9 (Sigma)
SpyCas9v3 (IDT)
HF1 SpyCas9 (NEB)
Ultrapure
Quick purification protocol
Commercial providers
Ultrapure
Quick purification protocol
Commercial providers
Without additional annealing step in preparation of gRNA complex
With additional annealing step in preparation of gRNA complex
B
HDR & NHEJ efficiency (% of population)
0
20
40
60
80
100
0%
0%
29.8%
12.8%
23.8%
13.3%
16.7%
8.9%
10.4%
12.1%
42.1%
21.5%
39.9%
21.0%
31.9%
18.7%
15.9%
18.2%
mNeonGreen+
mCherry-
Negative control
WT SpyCas9 (repl. 1)
WT SpyCas9 (repl. 2)
WT SpyCas9
WT SpyCas9v2 (Thermo)
WT SpyCas9 (repl. 1)
WT SpyCas9 (repl. 2)
WT SpyCas9
WT SpyCas9v2 (Thermo)
Ultrapure
Quick purification protocol
Commercial providers
Ultrapure
Quick purification protocol
Commercial providers
Without additional annealing step in preparation of gRNA complex
With additional annealing step in preparation of gRNA complex

(Previous page) Appendix Figure S2. Knock-out and knock-in efficiencies of different SpyCas9 proteins with and without pre-annealing gRNA complex. *D. discoideum* AX2 *act5::mCherry* cells were transfected with (A) 2.4μM donor oligos (37nt insertion/28nt homology arms) or (B) 10μg of *mNeonGreen-P2A* donor PCR product targeting aa9 of the *mCherry* gene. (A) Knock-out efficiencies with different purification methods of SpyCas9 (a 3-day protocol to produce an ultrapure extract or a quick one-day protocol) and different versions of SpyCas9 (WT, HiFi and HF1). For ultrapure WT SpyCas9 three biological replicates are shown, using separate cell batches. Knock-out efficiencies of equivalent commercially available protein extracts, including TrueCut Cas9 Protein v2 (Thermo Fisher A36498), Cas9 Protein (Sigma-Aldrich CAS9PROT-50UG), Alt-S.p. Cas9 Nuclease V3 (Integrated DNA Technologies (IDT) 1081058) and EnGen Spy Cas9 HF1 (New England Biolabs (NEB) M0667M) are also shown. (B) Knock-in efficiencies with different purification methods of SpyCas9 (a 3-day protocol to produce an ultrapure extract or a quick one-day protocol) and TrueCut Cas9 Protein v2 (Thermo Fisher A36498). For both (A) and (B), editing efficiencies are shown with (right) and without (left) pre-annealing step of the gRNA complex (see Methods).

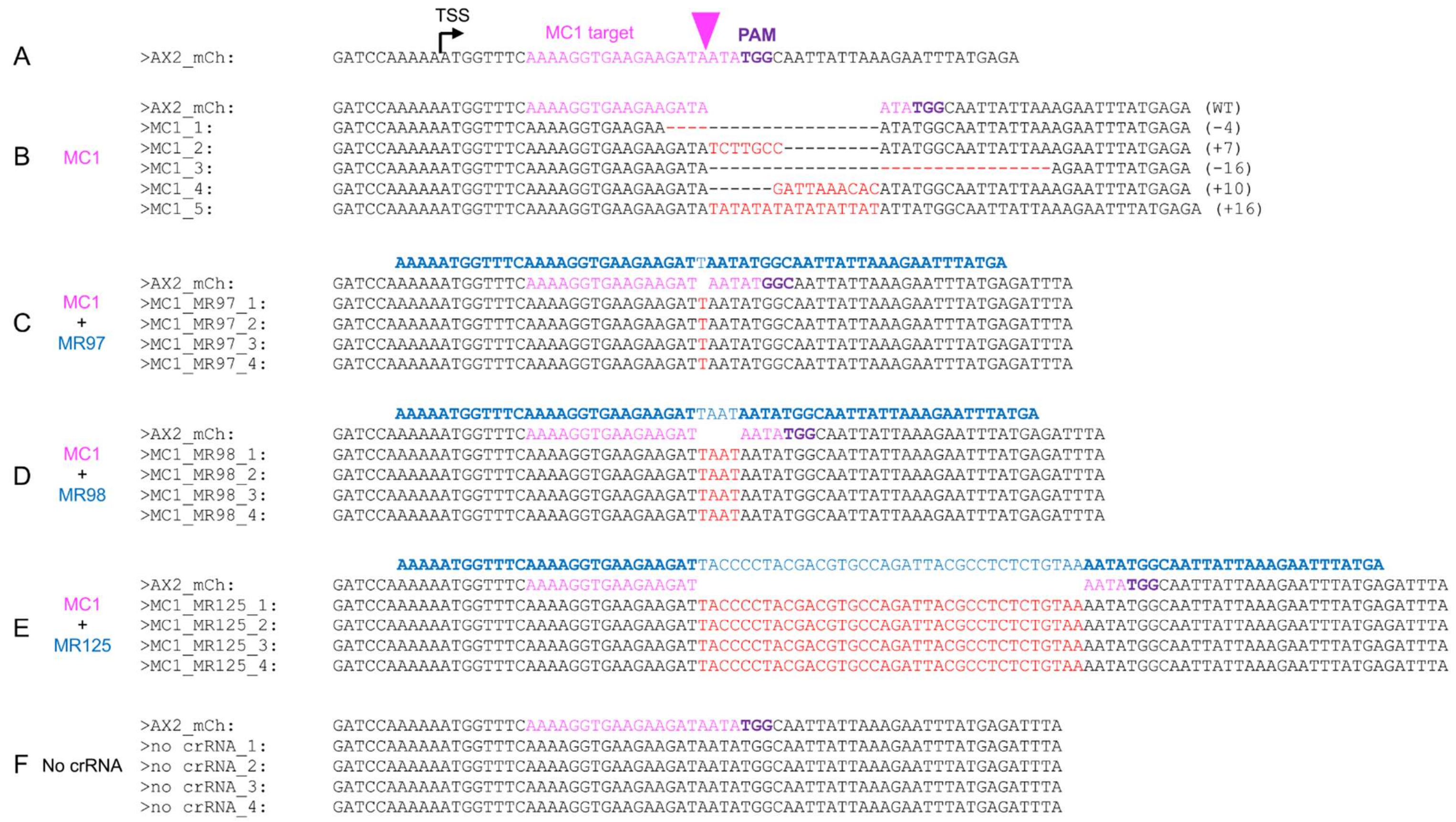

Appendix Figure S3. Aligned Sanger sequencing results of knock-out mutants. (A) Sequence of the *act5::mCherry* locus showing the target sequence (magenta), predicted cleavage site (magenta triangle) and downstream PAM (bold dark magenta) sequence. Sanger sequencing results (File S7) for different clones of *D. discoideum* AX2 *act5::mCherry* transfected with SpyCas9, tracrRNA and crRNA (MC1 targeting the aa9 of *mCherry* CDS) (C) MC1 crRNA and donor oligo (blue) for 1bp insertion/28bp homology arms (MR97), (D) MC1 crRNA and donor oligo for 4bp insertion/28bp homology arms MR(98), (E) MC1 crRNA and donor oligo for 37bp insertion/28bp homology arms (MR125), or as control (F) no crRNA and no donor oligo. Indels are shown in red. See Appendix Table S5 for MC1 crRNA spacer sequence. Donor oligo sequences are shown in blue, and can also be found in Appendix Table S6.

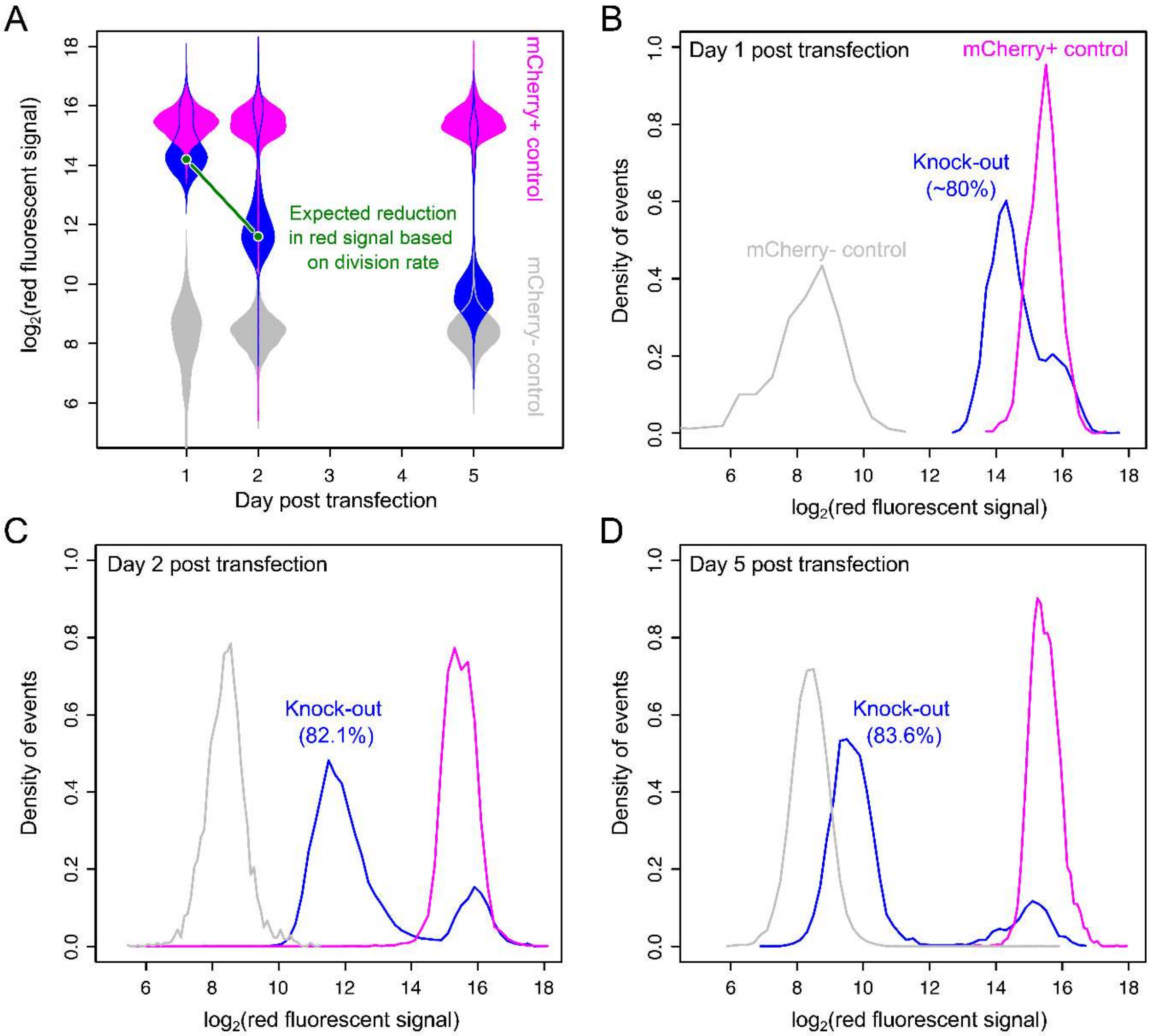

Appendix Figure S4. Quantification of knock-out mutants through flow cytometry. Knock-outs are produced by targeting amino acid 9 of *mCherry* CDS of the *D. discoideum* AX2 *act5*::*mCherry* strain with 2.4μM donor oligo (37nt insertion/28nt homology arms) without pre-annealing the gRNA complex. (A) Distribution of red fluorescent signal in CRISPR-Cas9 edited cells (blue), mCherry+ (magenta, *D. discoideum* AX2 *act5*::*mCherry*) and mCherry- (grey, *D. discoideum* AX2) control cells. Red fluorescent signal distribution is bimodal for CRISPR-Cas9 edited cells: some cells obtained knock-out mutation and therefore gradually lose fluorescent signal, whereas other cells were not edited and retain their fluorescent signal. Green line shows expected reduction in red fluorescent signal based on the expected dilution of mCherry protein per cell through cell division. The expected reduction perfectly follows the observed reduction. Distribution of red fluorescent signal for CRISPR-Cas9 edited cells, mCherry+ and mCherry- control cells (B) 1 day, (C) 2 days and (D) 5 days post-infection. Knock-out cells can be identified as soon as one day post-transfection, making it possible to isolate them after just one day.

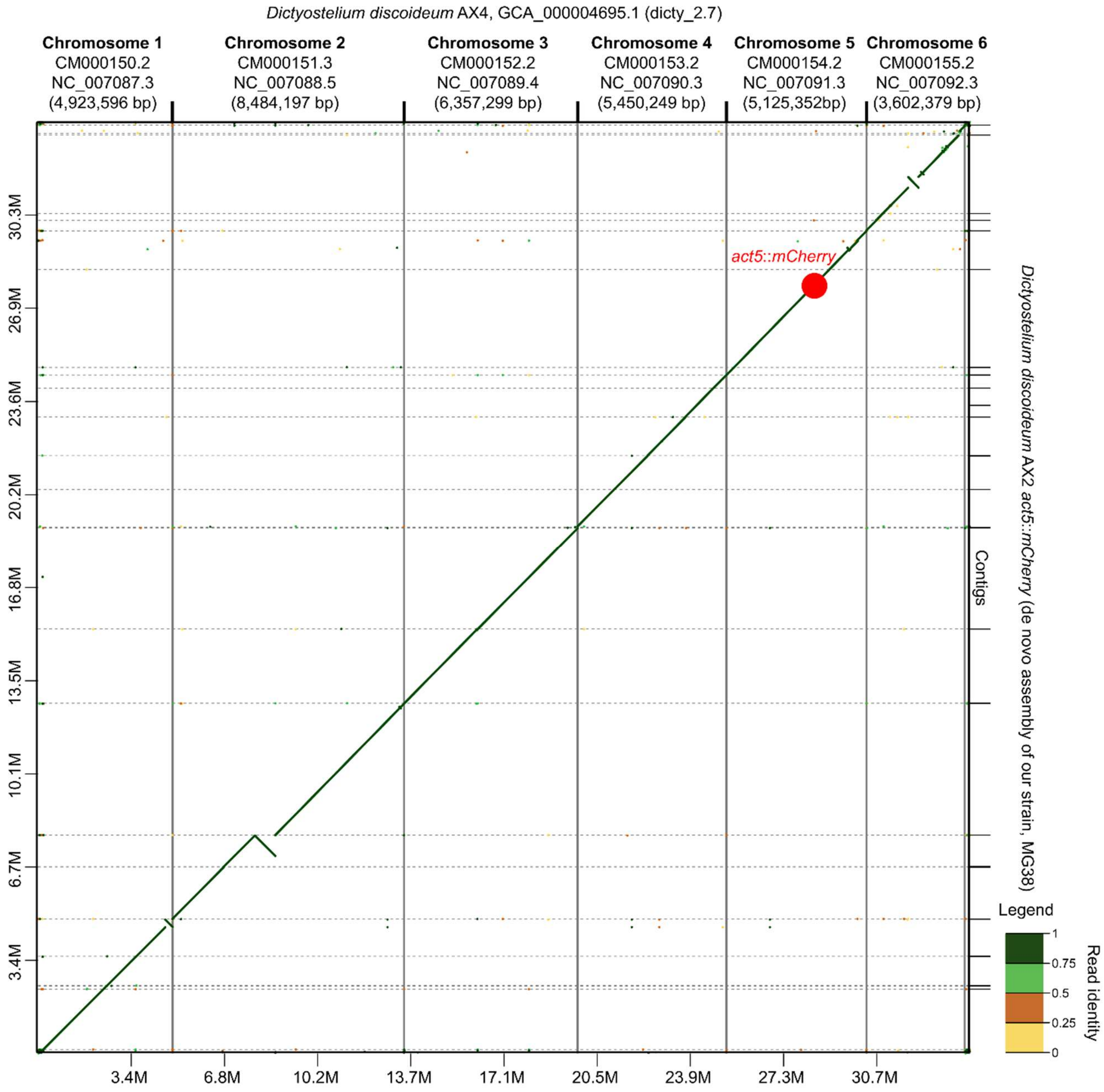

Appendix Figure S5. Comparison between *de novo* assembly of our *D. discoideum* AX2 *act5*::*mCherry* strain and the *D. discoideum* AX4 RefSeq strain. Mapping between chromosome-level assembly of *D. discoideum* AX4 (GCA_000004695.1) (x-axis) and our *de novo D. discoideum* AX2 *act5::mCherry* assembly (y-axis; MG38). For our *de novo* assembly, nanopore sequence data was pooled for all strains with an *act5*::*mCherry* integration site (Appendix Table S3). Small dots show mapped reads. Color legend shows quality of mapping: dark green is high-quality mapping and yellow is low-quality mapping. Single large red dot shows *act5::mCherry* integration site.

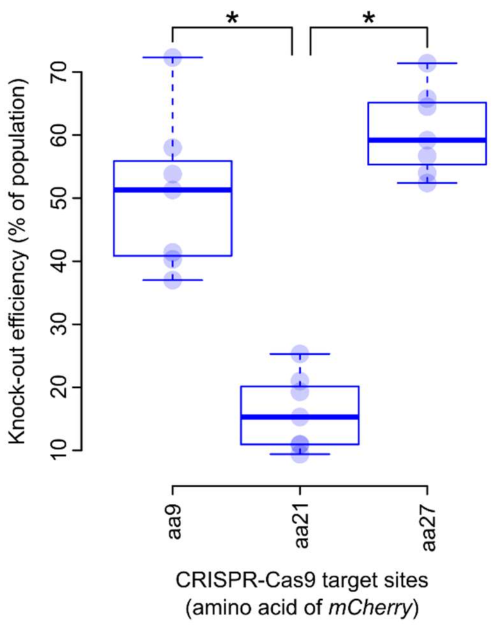

Appendix Figure S6. Knock-out efficiency for different CRISPR-Cas9 target sites in *mCherry*. *D. discoideum* AX2 *act5::mCherry* cells transfected with 2.4µM donor oligos (37nt insertion/28nt homology arms) targeting different sites of the *mCherry* gene, without pre-annealing the gRNA complex. Two of the three target sites show significantly higher knock-out efficiencies (pairwise Wilcoxon signed-rank test, $adjusted\ p < 0.01$). See Appendix Data S1 for data (https://doi.org/10.5281/zenodo.16919775).

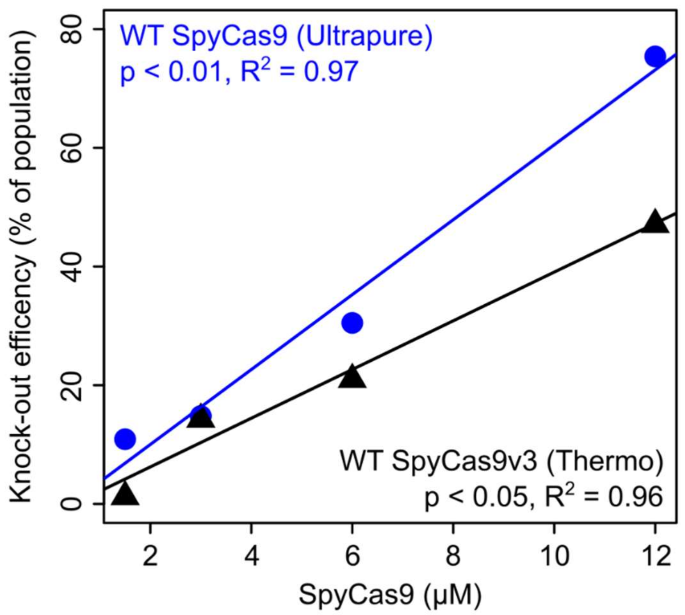

Appendix Figure S7. Effect of SpyCas9 concentrations on editing efficiency. *D. discoideum* AX2 *act5::mCherry* cells were transfected with 2.4μM donor oligos (37nt insertion/28nt homology arms) targeting aa9 of the *mCherry* gene. Knock-out efficiencies increase with higher SpyCas9 concentration for both Ultrapure WT SpyCas9 and WT SpyCas9v3 (Thermo). We expect that even higher knock-out efficiencies could be obtained with SpyCas9 concentrations above 12μM, but one has to be cautious about potential off-target edits.

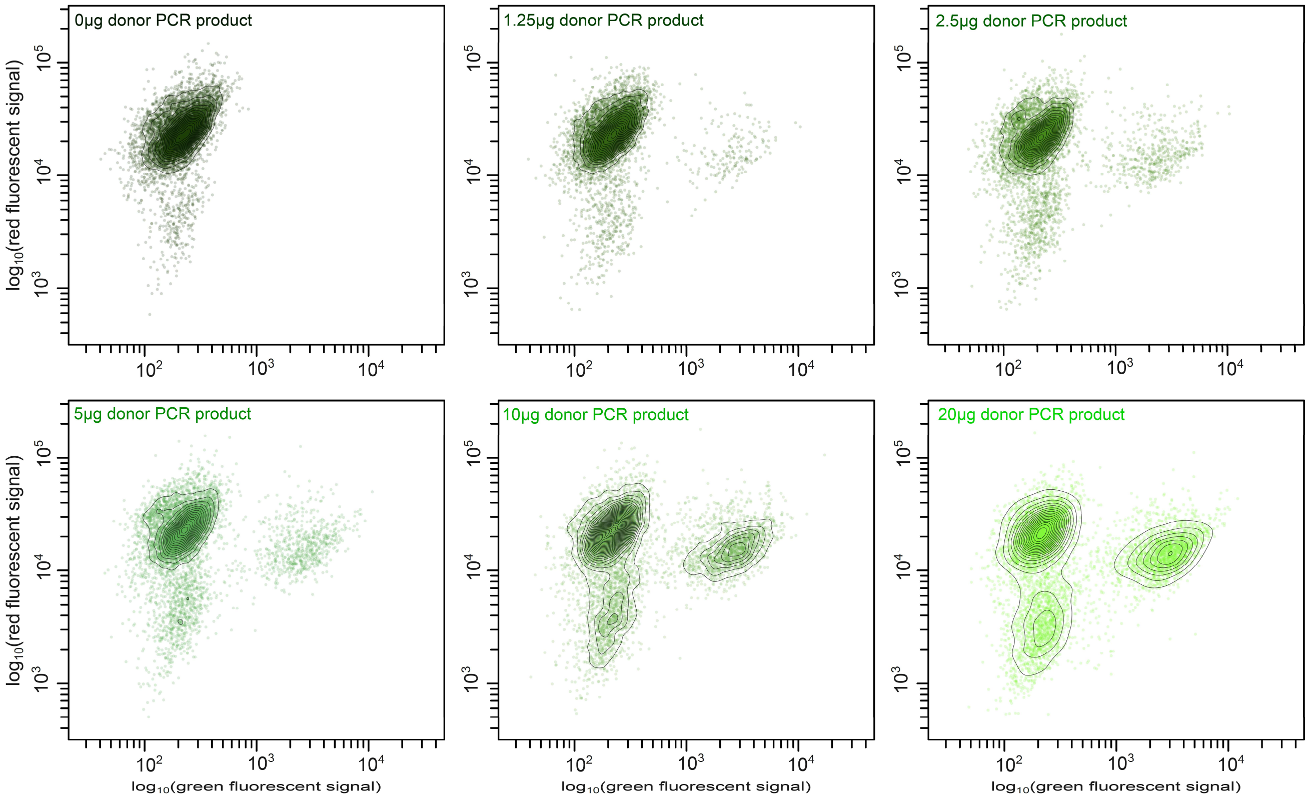

Appendix Figure S8. Flow cytometry data of knock-in experiments with different concentrations of donor PCR products. Knock-ins are produced by targeting amino acid 9 of *mCherry* CDS of *D. discoideum* AX2 *act5*::*mCherry* and a donor PCR product encoding for mNeonGreen-P2A in frame with mCherry (Figure 2A) without pre-annealing the gRNA complex. The same target and donor PCR product is used for all knock-ins experiments hereafter, unless noted otherwise. Flow cytometry results identical to those in Figure 2B, but separated by the quantity of donor PCR product added to the transfection: 0, 1.25, 2.5, 5, 10 or 20µg (0, 2.26, 4.52, 9.03, 18.06 or 36.12pmol respectively). Color codes correspond to Figure 2B. Contours indicate density of data points.

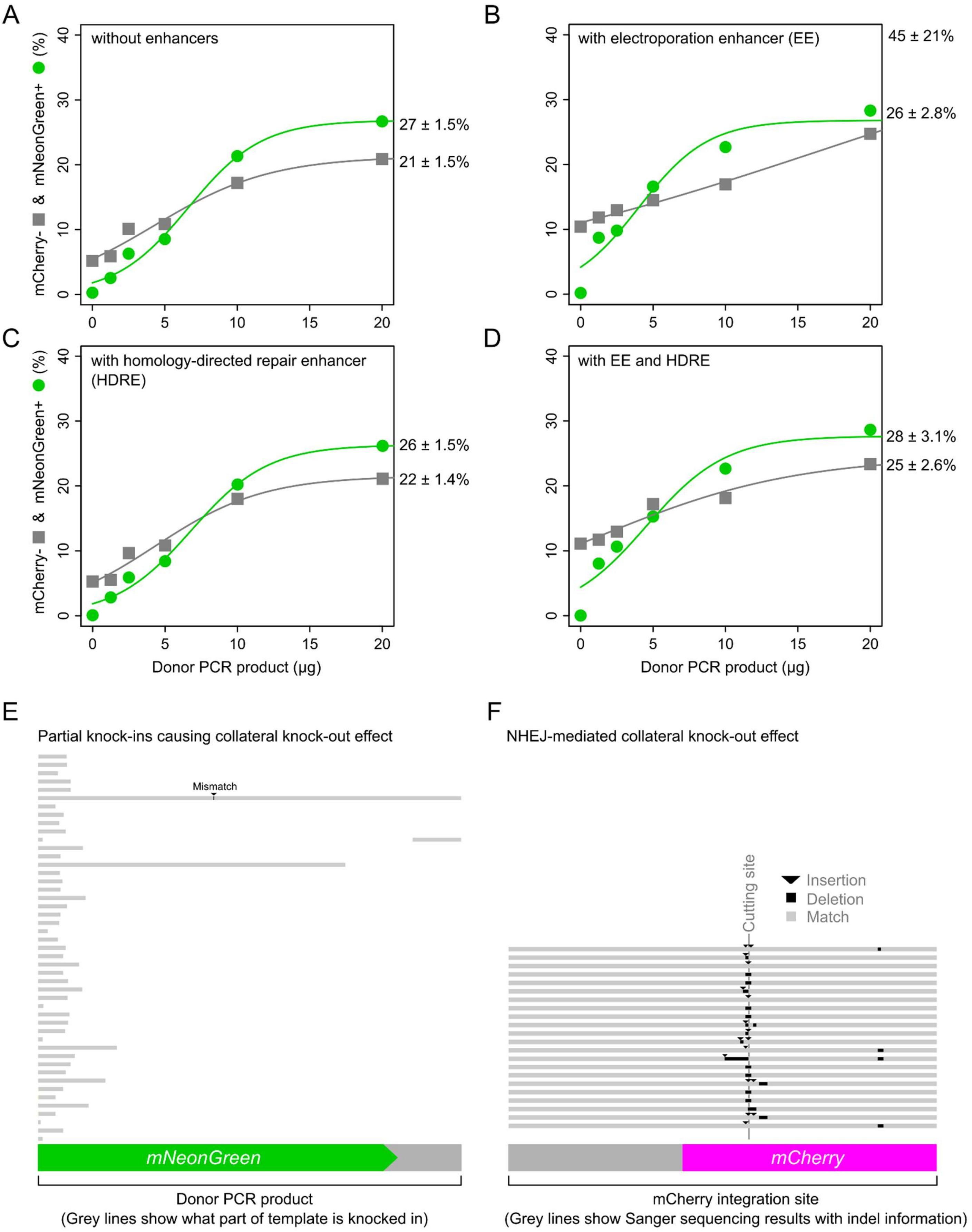
A
without enhancers
27 ± 1.5%
21 ± 1.5%
mCherry- & mNeonGreen+ (%)
B
with electroporation enhancer (EE)
45 ± 21%
26 ± 2.8%
C
with homology-directed repair enhancer (HDRE)
26 ± 1.5%
22 ± 1.4%
D
with EE and HDRE
28 ± 3.1%
25 ± 2.6%
Donor PCR product (µg)
E
Partial knock-ins causing collateral knock-out effect
Mismatch
mNeonGreen
Donor PCR product
(Grey lines show what part of template is knocked in)
F
NHEJ-mediated collateral knock-out effect
Cutting site
Insertion
Deletion
Match
mCherry
mCherry integration site
(Grey lines show Sanger sequencing results with indel information)

(Previous page) Appendix Figure S9. CRISPR-Cas9 mediated knock-ins. *D. discoideum* AX2 *act5::mCherry* was transfected with the RNP complex targeting *mCherry* (34/37nt homology arms) without pre-annealing the gRNA complex, using 0-20µg of a donor PCR product (*mNeonGreen-P2A*). mNeonGreen+ (green) and collateral mCherry- (grey) percentages (A) without enhancers, (B) with electroporation enhancer (EE; final concentration at 1.2µM in the transfection mix), (C) with homology-directed repair enhancer (HDRE; final concentration at 1.2µM in growth media), and (D) with EE and HDRE. There was no effect of the HDRE on knock-in and collateral knock-out probabilities, whereas EE increased collateral knock-out rates. Percentages show maximum knock-out and knock-in efficiencies and standard errors estimated using a sigmoidal fit to the data. See also Figure 2 and Appendix Data S1 for data. Collateral knock-outs are either caused by partial integration of the *mNeonGreen* donor PCR product (E) or by NHEJ (F). Partial integration of donor PCR product was biased to the 5' end of the PCR product (E), suggesting that integration was mediated by HDR with full-length left homology to the target site and a truncated right homology, likely mediated by small sequence similarities between the CDS of *mNeonGreen* and *mCherry*. For Sanger sequencing data see File S7.

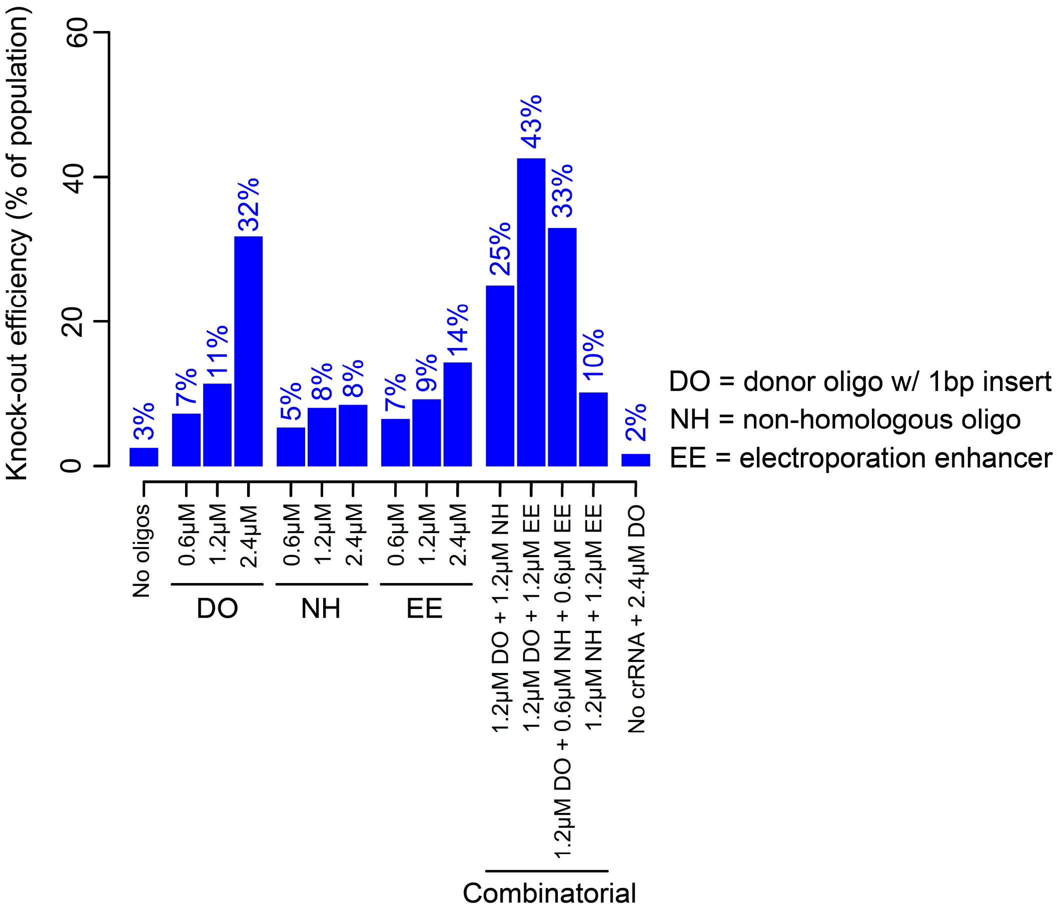

Appendix Figure S10. Quantification of secondary knock-out effect using donor oligos (DO), non-homologous oligos (NH) and electroporation enhancer (EE). *D. discoideum* AX2 *act5::mCherry* was transfected with the RNP complex targeting *mCherry* without pre-annealing the gRNA complex. Combinations of donor oligo (1bp insertion and 28/31bp left/right homology arms), NH 60nt oligo (Appendix Table S6), and EE (IDT) are added to discern the impact of donor oligos on homology-directed repair (HDR) and the carrier effect. Increasing amounts of donor oligo improves knock-out efficiencies. Addition of NH oligos and EE improves knock-out efficiency (8% and 14% maximum knock-out efficiency, respectively, compared to 3% knock-out efficiency without oligo). Combinations of donor oligo at 1.2µM with NH oligo, EE or NH+EE (final combined concentration of 2.4µM) further enhances knock-out efficiencies of the donor oligo (25%, 43% and 33%, respectively, compared to 11% knock-out efficiency with 1.2µM donor oligo alone), suggesting a carrier effect.

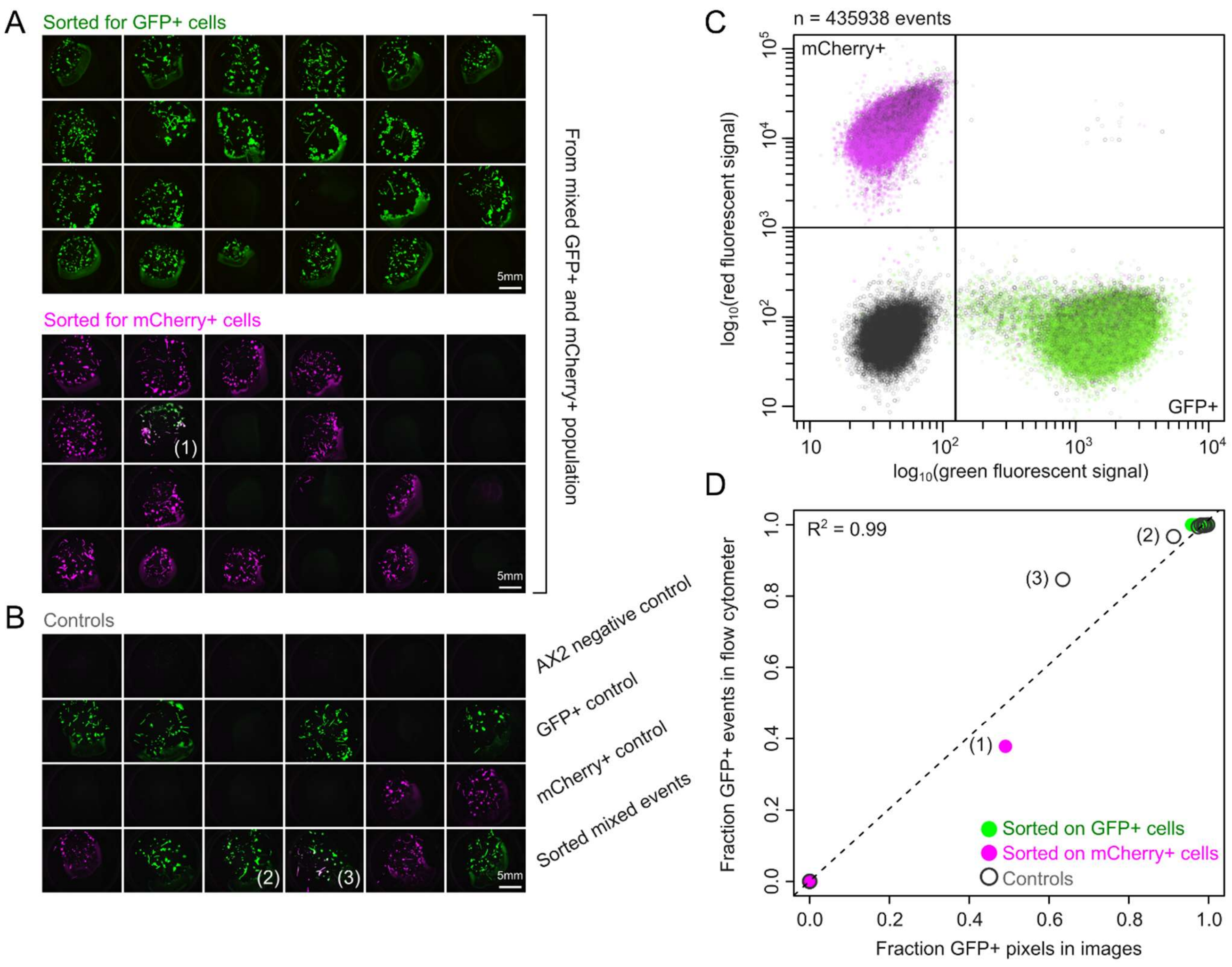

Appendix Figure S11. Quantification of sorting accuracy using a combination of microscopy and flowcytometry. (A) 24-well plates after sorting mixed population of *D. discoideum* AX2 mCherry+/GFP+ cells. Upper plate sorted for GFP+ cells and lower plate for mCherry+ cells. Plates are identical to those shown in Figure 4C-D. Cells were grown for 4 days after sorting, leaving sufficient time for cells to form plaques. (B) 24-well control plate where we sorted single cells from clonal populations of non-fluorescent *D. discoideum* AX2, *D. discoideum* AX2 *act15*::*gfp* or *D. discoideum* AX2 *act5::mCherry*, as negative controls, and mixed events (with GFP+ and mCherry+ signal) from a mixed population of *D. discoideum* AX2 *act15::gfp* and *D. discoideum* AX2 *act5::mCherry*, as a positive control for mixing. For each well, we also collected several sori and analyzed cells through flow cytometry to quantify percentage of GFP+ and mCherry+ cells. (C) Combined flow cytometry results from all wells: events from plate with GFP+ sorted cells shown in green, events from plate with mCherry+ sorted cells shown in magenta and events from control plate shown in grey. (D) Correlation between fraction of GFP+ pixels in microscopy images and GFP+ events in flow cytometry ($R^2 = 0.99, p < 0.001$). From the GFP+ and mCherry+ sorted plates only a single well showed mixed cells (1), while the other mixed wells, (2) and (3), were part of the positive controls in (B).

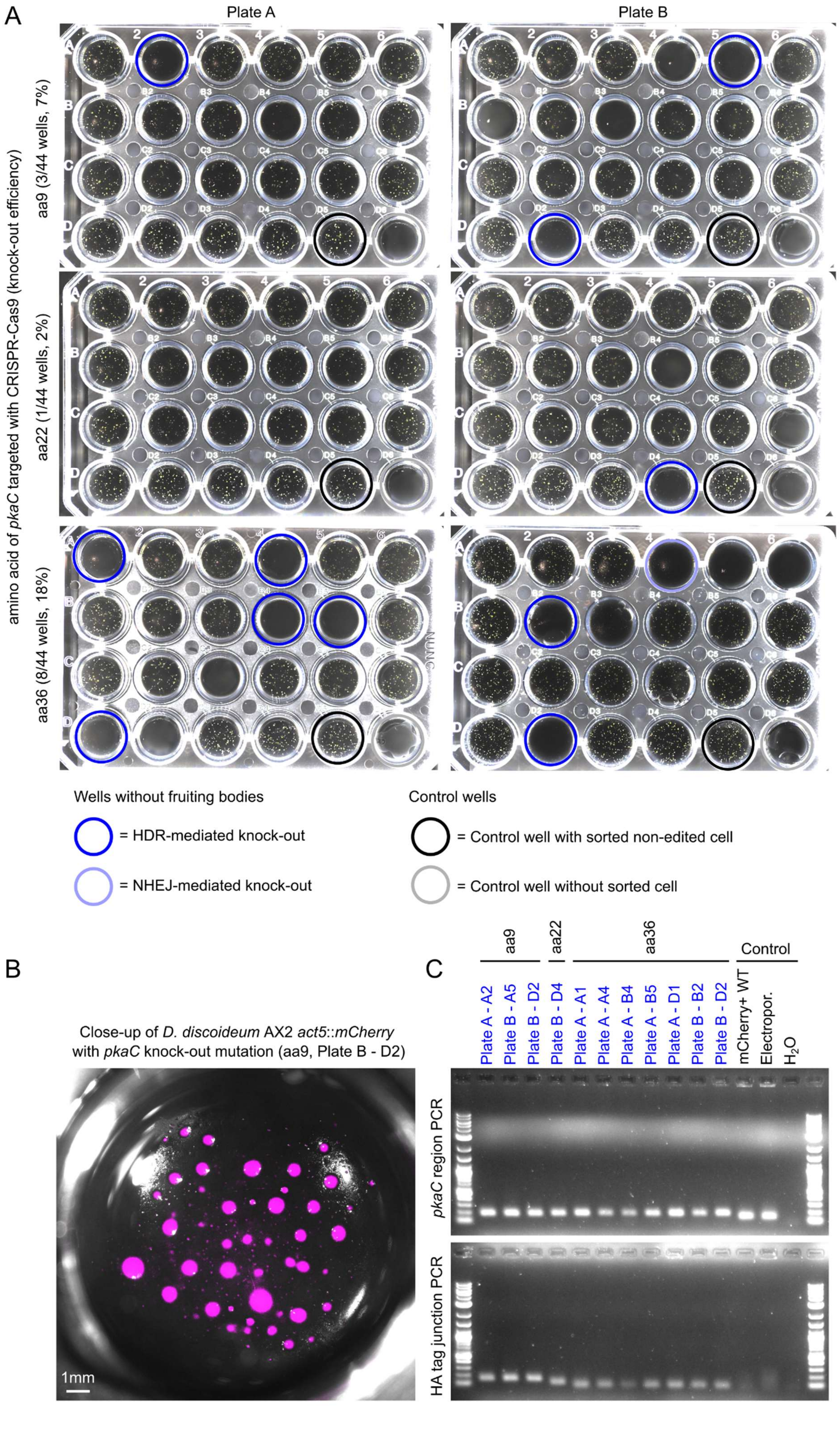

A
Plate A
Plate B
amino acid of pkaC targeted with CRISPR-Cas9 (knock-out efficiency)
aa9 (3/44 wells, 7%)
aa22 (1/44 wells, 2%)
aa36 (8/44 wells, 18%)
Wells without fruiting bodies
= HDR-mediated knock-out
= NHEJ-mediated knock-out
Control wells
= Control well with sorted non-edited cell
= Control well without sorted cell
B
Close-up of D. discoideum AX2 act5::mCherry with pkaC knock-out mutation (aa9, Plate B - D2)
1mm
C
aa9
aa22
aa36
Control
Plate A - A2
Plate B - A5
Plate B - D2
Plate B - D4
Plate A - A1
Plate A - A4
Plate A - B4
Plate A - B5
Plate A - D1
Plate B - B2
Plate B - D2
mCherry+ WT
Electropor.
H2O
pkaC region PCR
HA tag junction PCR

(Previous page) Appendix Figure S12. CRISPR-Cas9 knock-outs targeting *pkaC*. (A) Sorted plates after transfection of *D. discoideum* AX2 *act5::mCherry* cells with CRISPR-Cas9 targeting amino acid 9 (aa9), 22 (aa22) or 36 (aa36) of *pkaC* CDS with donor oligos (37bp insertion/28bp homology arms) generating knock-out mutations. Wells with blue circles lack fruiting body development, but do show cells at the mound stage, suggestive of *pkaC* knock-out. (B) Example of well with *pkaC* knock-out (also shown in Figure 5A). (C) PCR of *pkaC* locus (primers MR237/238) around the CRISPR target sites and downstream junction PCR (primers MR129/MR238) confirming HDR-mediated knock-out of *pkaC* in respective wells. Knock-out efficiencies are highest (18%) for third (aa36) CRISPR-Cas9 target. See Appendix Table S2 for confirmation primers and File S7 for Sanger sequencing results.

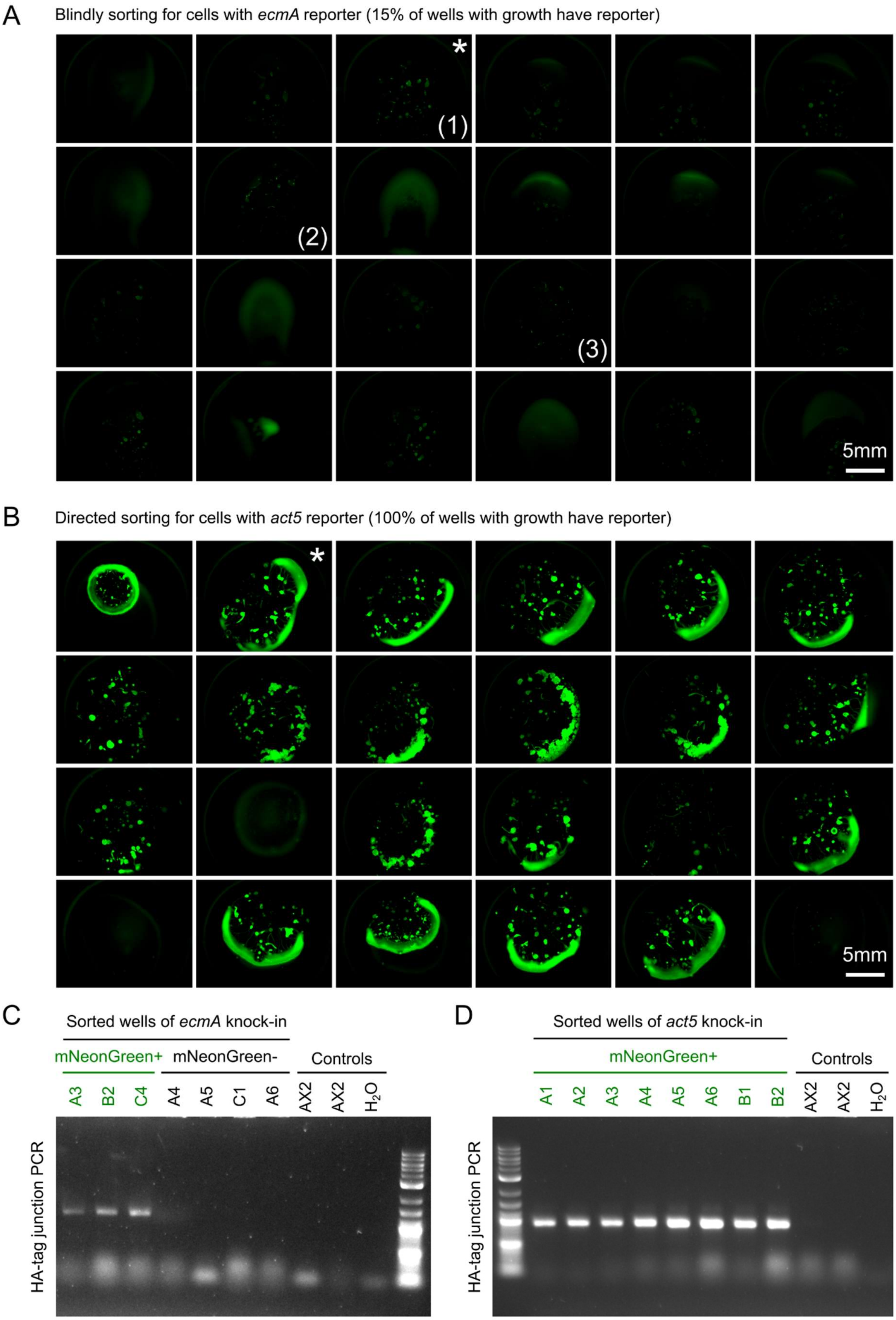
A
Blindly sorting for cells with *ecmA* reporter (15% of wells with growth have reporter)
*
(1)
(2)
(3)
5mm
B
Directed sorting for cells with *act5* reporter (100% of wells with growth have reporter)
*
5mm
C
Sorted wells of *ecmA* knock-in
mNeonGreen+
mNeonGreen-
Controls
A3 B2 C4 A4 A5 C1 A6 AX2 AX2 H_2O
HA-tag junction PCR
D
Sorted wells of *act5* knock-in
mNeonGreen+
Controls
A1 A2 A3 A4 A5 A6 B1 B2 AX2 AX2 H_2O
HA-tag junction PCR

(Previous page) Appendix Figure S13. Sorted plates and PCR confirmation of expression reporters. Fluorescence microscopy images of sorted plates after transfection for both the (A) *ecmA* and (B) *act5* expression reporters with SM5-charcoal-agar background and *E. coli* B/r (example wells are also shown in Figure 5B and 5C). For the *ecmA* expression reporter (*D. discoideum* AX2 *ecmA::mNeonGreen-P2A-ecmA*), cells were sorted blindly and three wells, numbered (1)-(3), showed *ecmA* expression in the anterior part of slugs (see also Figure 5). Well shown in Figure 5B is highlighted in (A) by an asterisk. For *act5* expression reporter (*D. discoideum* AX2 *act5::mNeonGreen-P2A-act5*), mNeonGreen+ cells were sorted directly after transfection, therefore all wells with growth show expected *act5* expression. Wells in (A) and (B) with no growth give a diffuse green fluorescence signal due to autofluorescence of the bacteria lawn. (C)-(D) Confirmation PCRs of *ecmA* and *act5* expression reporters, using junction PCRs with primer sets MR146/MR113 for *ecmA* and MR101/MR113 for *act5*. A PCR band is only observed in the case of a target specific integration. Cells were transfected without pre-annealing of the gRNA complex.

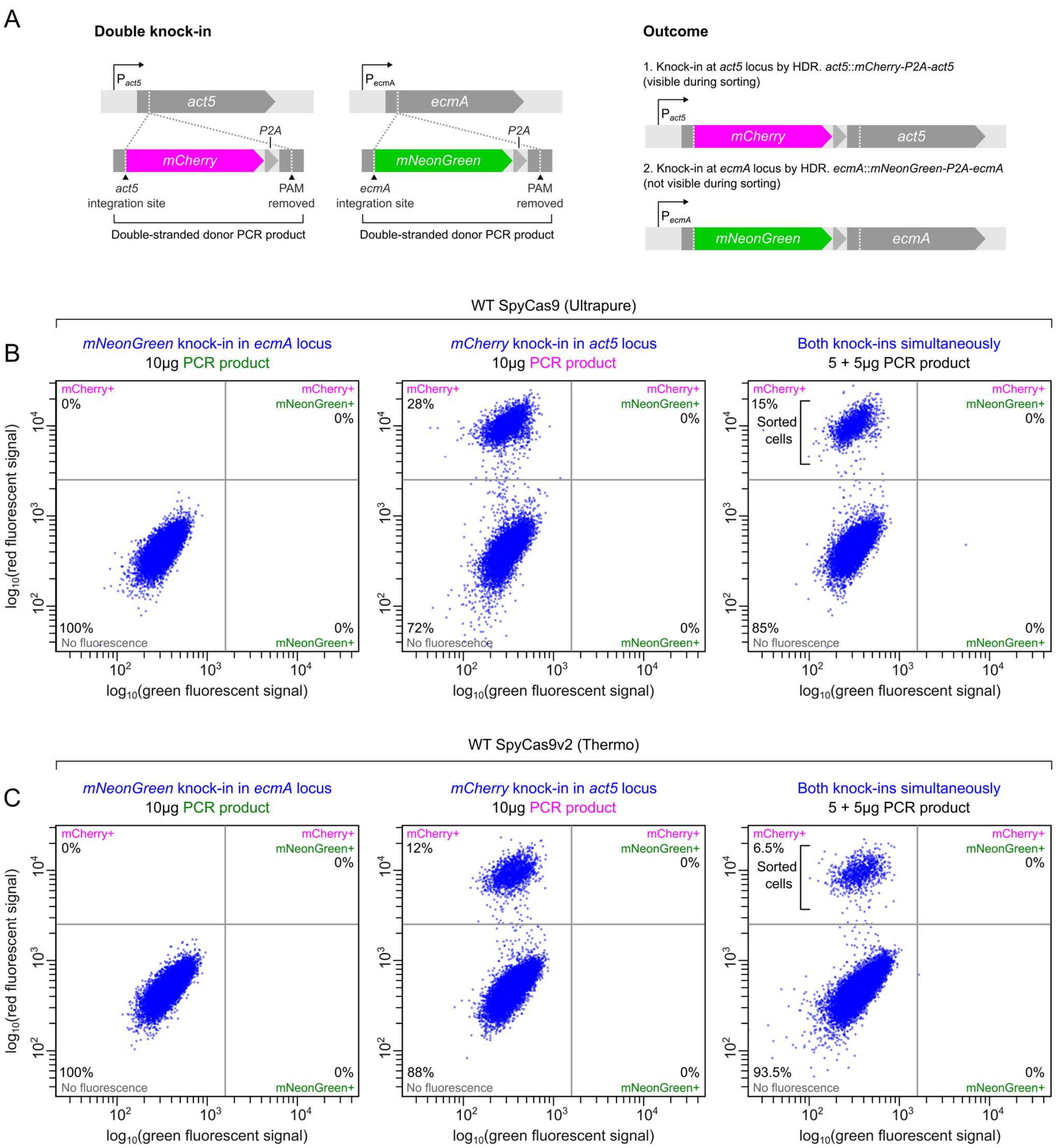

Appendix Figure S14. Simultaneous CRISPR-Cas9 editing of two loci by generating *act5* and *ecmA* double knock-in. (A) Double knock-in experiment in *D. discoideum* AX2 with *act5* (*act5*::*mCherry-P2A-act5*) and *ecmA* (*ecmA*::*mNeonGreen-P2A-ecmA*) expression reporters. Flow cytometry plots show results of *ecmA* knock-in only (left panel), *act5* knock-in only (middle panel), and double *act5* and *ecmA* knock-ins (right panel) with either (B) ultrapure WT SpyCas9 or (C) TrueCut Cas9 Protein v2 (Thermo Fisher A36498). Transfected cells were sorted for mCherry expression, as *ecmA* is expressed in the slug stage only (i.e., no mNeonGreen+ cells are present during sorting), and screened for double knock-ins using fluorescence microscopy (Appendix Figure S15). Flow cytometry results are acquired with the BD FACSAria Fusion Flow Cytometer for sorting.

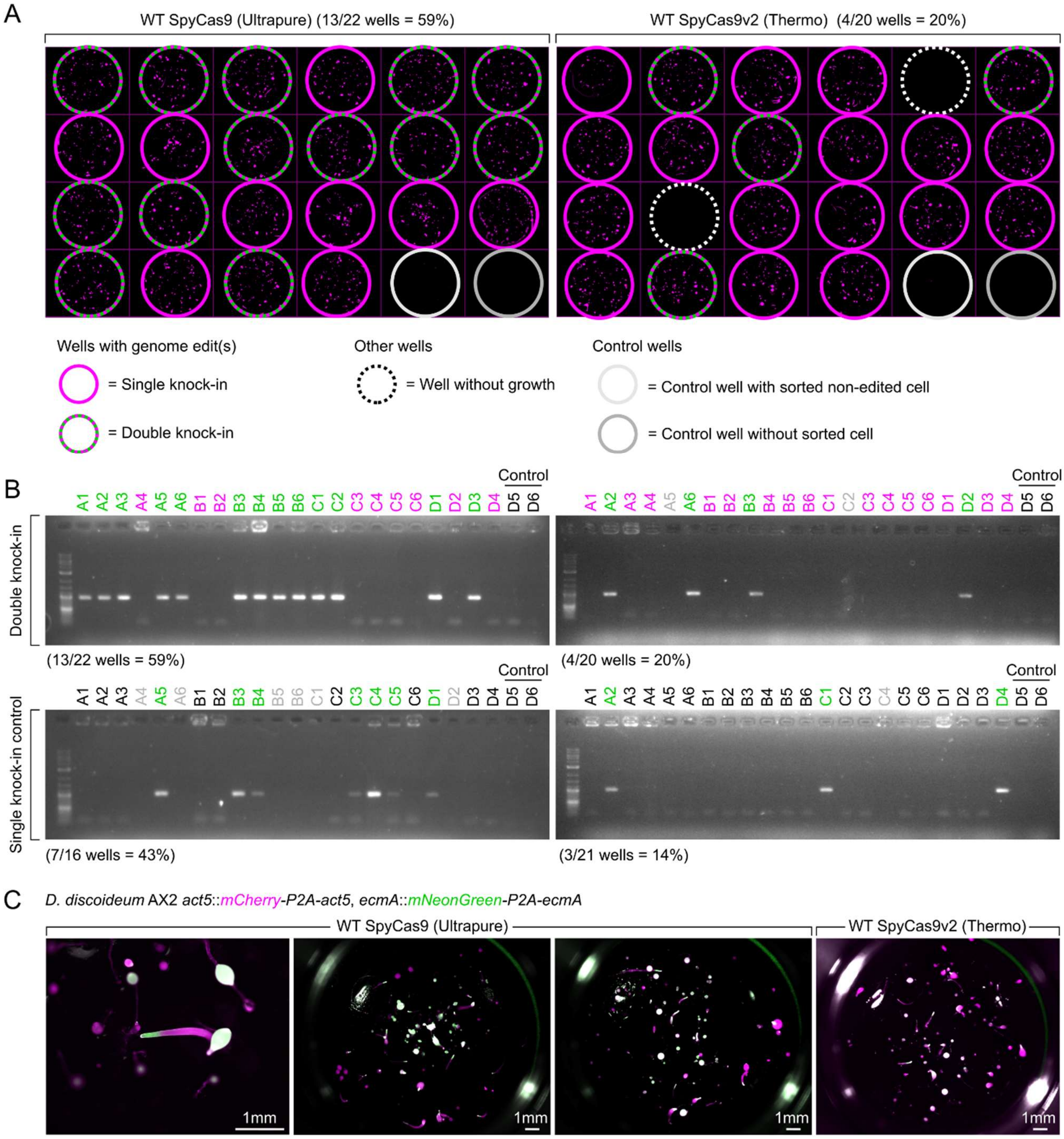

Appendix Figure S15. Post-transfection validation of double knock-in mutant. Double knock-in with *act5* (*act5*::*mCherry*-*P2A*-*act5*) and *ecmA* (*ecmA*::*mNeonGreen*-*P2A*-*ecmA*) expression reporters. Transfections were performed with either ultrapure WT SpyCas9 protein (left) or TrueCut Cas9 Protein v2 (Thermo Fisher A36498) (right). (A) Plates with mCherry+ sorted cells (see Appendix Figure S14). Colored circles show wells with single- (magenta) and double- (dashed magenta and green) gene edited cells. Dashed circles show wells without growth and grey circles show controls. (B) Confirmation of knock-ins at *ecmA* locus by PCR (primers MR243/MR245). For a fraction of mutants, knock-ins were also validated using Sanger sequencing (File S7). With exception of well A2 in the left upper gel, which showed a mixed clone, all Sanger sequencing results confirmed the

expected scarless integration of the donor PCR product. Knock-in efficiency at *ecmA* locus was higher for double knock-in experiment (59% and 20% for each SpyCas9 variant respectively), with mCherry+ sorted cells, than for single knock-in control (43% and 14% for each SpyCas9 variant respectively), with blindly sorted cells. (C) Example of slugs and wells with double knock-ins: *D. discoideum* AX2 *act5*::*mCherry-P2A-act5*, *ecmA*::*mNeonGreen-P2A-ecmA.*

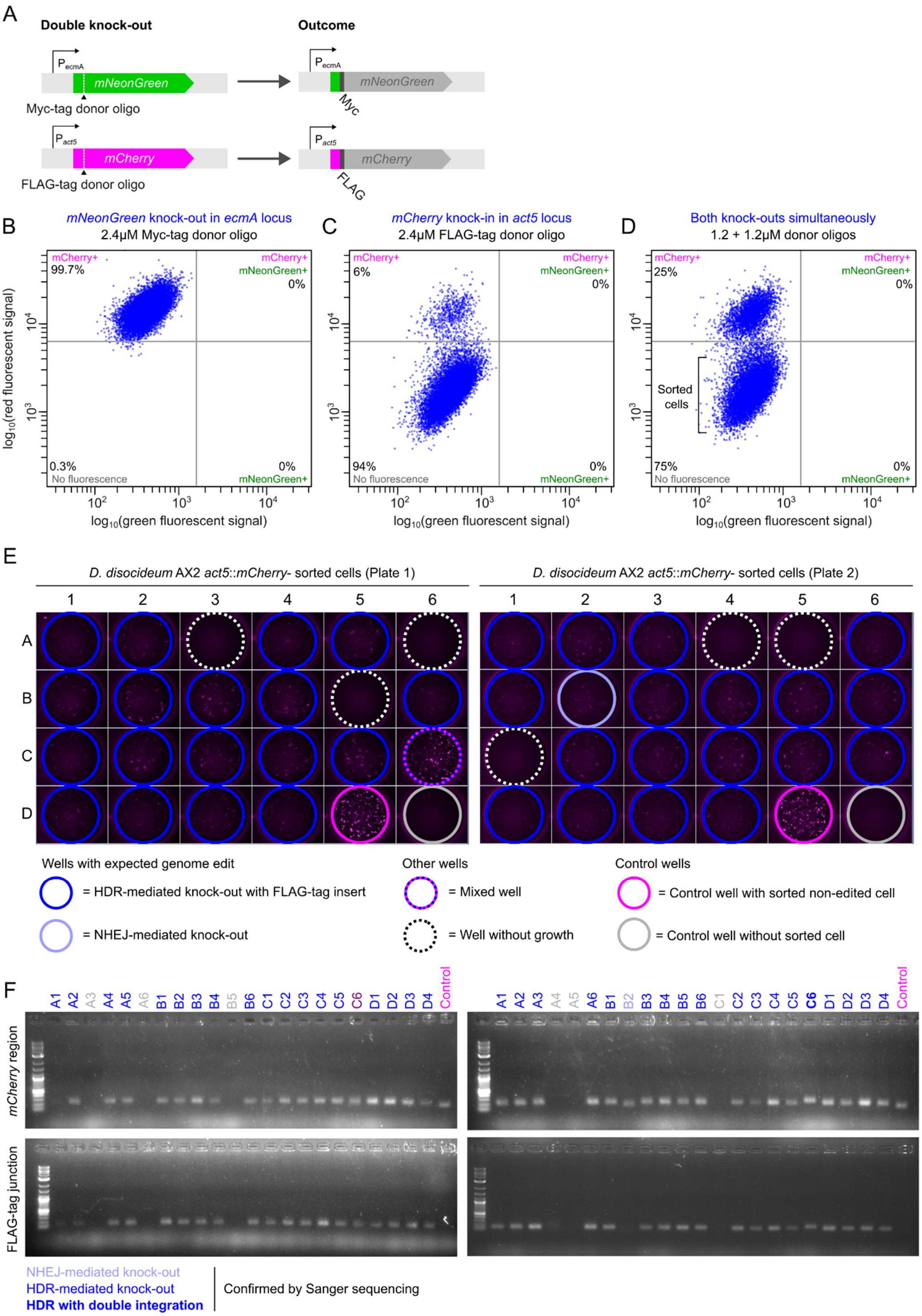
A
Double knock-out
Outcome
mNeonGreen
Myc-tag donor oligo
Myc
mCherry
FLAG-tag donor oligo
FLAG
B
mNeonGreen knock-out in ecmA locus
2.4µM Myc-tag donor oligo
C
mCherry knock-in in act5 locus
2.4µM FLAG-tag donor oligo
D
Both knock-outs simultaneously
1.2 + 1.2µM donor oligos
mCherry+
mCherry+ mNeonGreen+
No fluorescence
mNeonGreen+
99.7%
0.3%
6%
94%
25%
75%
0%
Sorted cells
$\log_{10}$(red fluorescent signal)
$\log_{10}$(green fluorescent signal)
E
D. disocideum AX2 act5::mCherry- sorted cells (Plate 1)
D. disocideum AX2 act5::mCherry- sorted cells (Plate 2)
Wells with expected genome edit
= HDR-mediated knock-out with FLAG-tag insert
= NHEJ-mediated knock-out
Other wells
= Mixed well
= Well without growth
Control wells
= Control well with sorted non-edited cell
= Control well without sorted cell
F
mCherry region
FLAG-tag junction
Control
NHEJ-mediated knock-out
HDR-mediated knock-out
HDR with double integration
Confirmed by Sanger sequencing

(Previous page) Appendix Figure S16. Double knock-out experiment targeting *D. discoideum* AX2 *act5*::*mCherry-P2A-act5* and *ecmA*::*mNeonGreen-P2A-ecmA*. (A) Schematic of HDR-mediated double knock-out experiment where *mCherry* (aa27) and *mNeonGreen* (aa30) are targeted by donor oligos with FLAG-tag and Myc-tag insert sequences respectively. The purpose of the experiment is to determine whether donor oligos can be wrongly integrated in double knock-out experiments. Since we can sort mCherry- cells after transfection we specifically examine whether all *mCherry* knock-out cells have the expected FLAG-tag integration and not the Myc-tag. (B)-(D) Flow cytometry results from single (controls) and double knock-out experiments. (E) Plates after sorting for mCherry- cells in double-knockout experiment. Blue circles show wells with mCherry- cells. Dashed circles show mixed well (blue and magenta dashes) and wells without growth (white). Magenta and grey circles highlight wells with non-edited ancestral cells, expressing *mCherry*, and wells without sorted cell. (F) Confirmation PCRs around target site (top panel, primers MR245/MR103) and at downstream junction (bottom panel, primers MR221/MR103) confirm that all HDR-mediated knock-out mutants have integrated the correct FLAG-tag and not the Myc-tag donor oligo. See also File S7 for Sanger sequencing results. One of the mutants had a double integration of the FLAG-tag donor oligo (well C6 of plate 2). One of the mutants had an NHEJ-mediated *mCherry* knock-out (well B2 of plate 2).

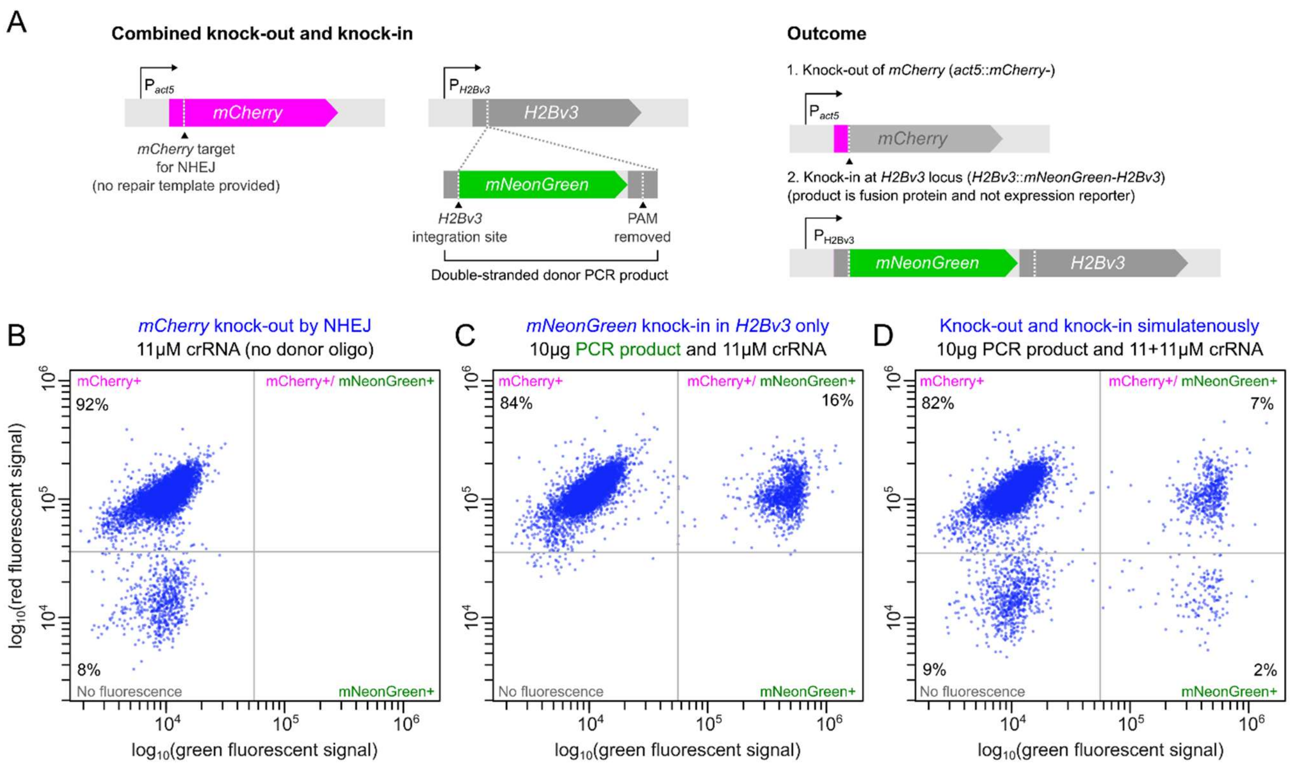

Appendix Figure S17. Simultaneous knock-out and knock-in CRISPR-Cas9 editing. (A) Simultaneous knock-out of *mCherry* (*act5*::*mCherry-*) and knock-in at *H2Bv3* (*H2Bv3*::*mNeonGreen-H2Bv3*) in *D. discoideum* AX2 *act5::mCherry* cells. Genome editing was performed without pre-annealing gRNA complex. Flow cytometry plots show results of (B) *mCherry* knock-out only, (C) *H2Bv3* knock-in only, and (D) a double knock-out and knock-in experiment. Cells with a knock-in had a significantly higher probability of also having a knock-out mutation: 22% (2 out of the 9% knock-in cells) instead of 10% (9 out of the 91% wild-type cells). Conversely, cells with a knock-out had a higher probability of also having a knock-in mutation: 18% (2 out of the 11% knock-out cells) instead of 8% (7 out of the 89% wild-type cells) (Fisher's Exact Test; $p < 10^{-10}$, odds ratio=2.3 with [1.8-2.8] 95%-confidence intervals, n=7223 cells). Flow cytometry results are obtained with the BD FACSymphony A3 analyzer.

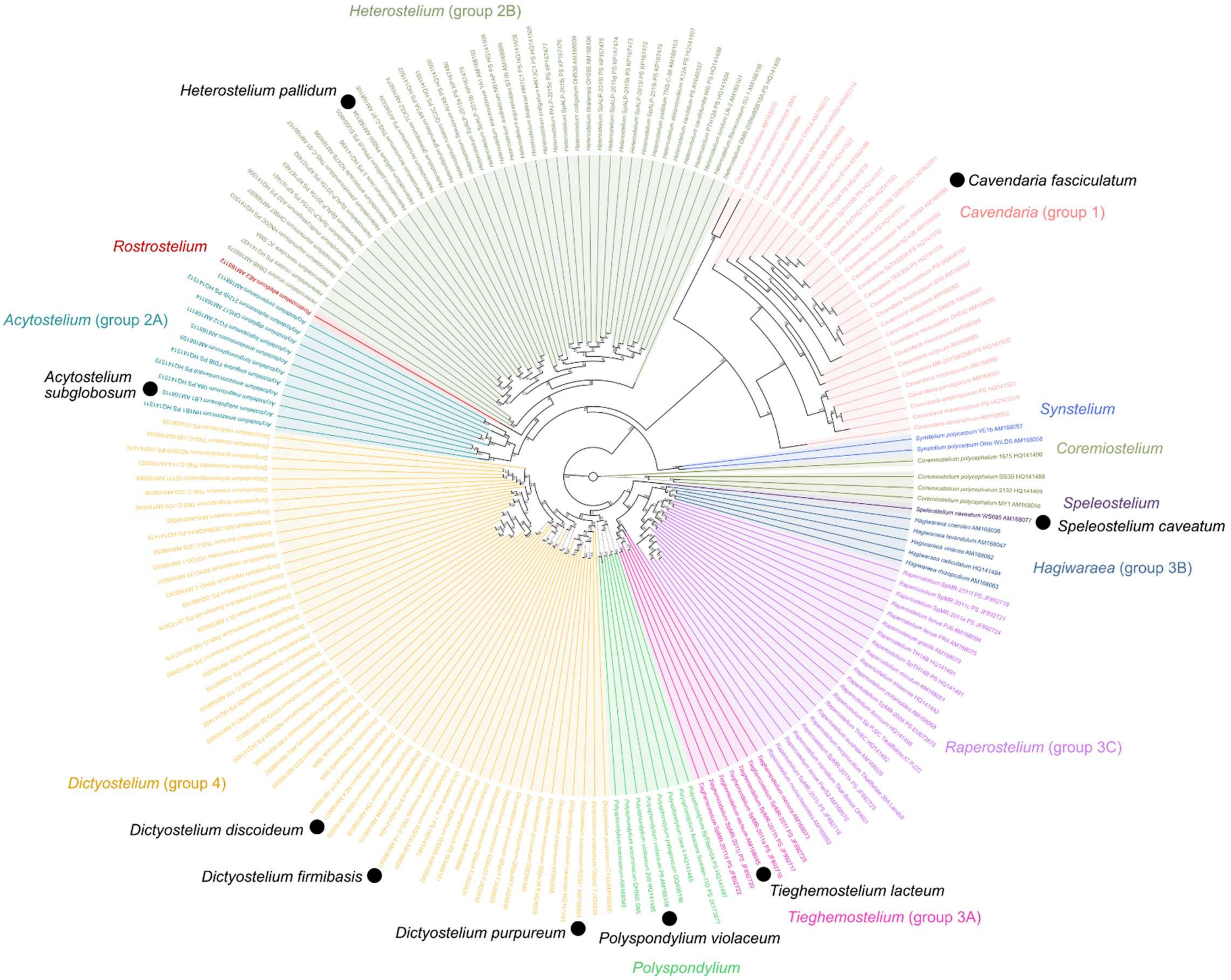

Appendix Figure S18. Phylogenetic tree with representative *Dictyostelid* species. Phylogenetic tree is based on multiple-sequence alignment of 18S rDNA of *Dictyostelids* as provided by (Sheikh *et al*, 2018). Colors in phylogenetic tree highlight *Dictyostelid* families and correspond to those in Figure 7A.

Captions for Appendix Files

All Appendix Files (File S1-S8) are deposited to Zenodo (https://doi.org/10.5281/zenodo.16919775)

Appendix File S1. Plasmid maps. Snapgene files with plasmid maps for pMG005 with codon-optimized *mNeonGreen* and pMG008 with codon-optimized *mCherry*, as well as Snapgene files for pHO4d-Cas9, pET-FLAG-SpCas9-HF1 and pET-HiFi SpCas9-NLS-6xHis with SpyCas9 variants (see Appendix Figure S2).

Appendix File S2. Time-lapse of strains with *ecmA* and *act5* expression reporters in Figure 5. Time-lapse video of feeding front and fruiting body development for (left) *D. discoideum* AX2, (middle) *ecmA* reporter and (right) *act5* reporter strains. *ecmA* reporter shows clear induction of *ecmA* expression at anterior part of slugs, while *act5* is expressed throughout the slugs.

Appendix File S3. Raw image data of slugs in Figure 5. Images show *ecmA* expression in slugs (z-stacks) and csv files show raw data for *ecmA* expression profiles along anterior-posterior axis of slugs.

Appendix File S4. Raw image data of cells in Figure 6. Images show maximal projection of *act5* expression in cells as well as cell masks and csv files provide raw pixel intensities from cell periphery to center.

Appendix File S5. Videos of *Dictyotselid* species (low zoom). Overview time-lapse movies of WT and *act5*::*mNeonGreen-P2A-act5* knock-in strains of *D. discoideum*, *D. firmibasis*, *D. purpureum*, *P. violaceum*, *T. lacteum* and *H. pallidum*. For all species, growth of knock-in mutants (top) and WT (bottom) were similar: left panels show combined green fluorescence and spotlight image (using same color scale as in Figure 7); middle panels show green fluorescence only and right panels show spot lights only. Scale bars equal 5mm.

Appendix File S6. Videos of *Dictyostelid* species (high zoom). Time-lapse movies at high magnification of *act5*::*mNeonGreen-P2A-act5* knock-in strains of *D. discoideum*, *D. firmibasis*, *D. purpureum*, *P. violaceum*, *T. lacteum* and *H. pallidum* (using same color scale as in Figure 7). Frames highlighted in Figure 7 are included as image files. Scale bars are equal to 1mm.

Appendix File S7. Sanger sequencing data. Sequencing files (AB1) of confirmation PCR products sequenced by Eurofins or Eurofins for Figure 7, and Appendix Figures S3, S9, S12, S15 and S16.

Appendix File S8. Ungated flow cytometry data. Flow cytometry files (FCS) for Figures 1, 2, 3, 6, 7 obtained with either the BD FACSymphony A3 analyzer or BD FACSAria Fusion Flow Cytometer.

Appendix Table S1. Strain list.

Species / strain	Resource ID	Resource
Escherichia coli B/r	DBS0305924	dictyBase
Dictyostelium discoideum AX2 (Kay lab)	DBS0235521	dictyBase
Dictyostelium discoideum AX2 *act15*::*gfp*	DBS0235536	dictyBase
Dictyostelium discoideum AX2 *act5*::*mCherry, Hyg*	MG38	This study[1]
Dictyostelium discoideum NC4	DBS0304666	dictyBase
Dictyostelium discoideum NC4 *act5*::*mCherry, Hyg*	HM1912	(Paschke *et al*, 2018)
Dictyostelium firmibasis TNS-C-14	DBS0235812	dictyBase
Dictyostelium purpureum WS321	DBS0235881	dictyBase
Polysphondylium violaceum P6, S209	DBS0236814	dictyBase
Tieghemostelium lacteum S561	DBS0235831	dictyBase
Acytostelium subglobosum LB1	DBS0235452	dictyBase
Heterostelium pallidum PN500	DBS0302501	dictyBase
Cavendaria fasciculatum Swok OW9A	DBS0235811	dictyBase
Speleostelium caveatum	DBS0235736	dictyBase

Appendix Table S2. List of generated knock-out strains.

Identifier	Species / strain	Target gene / aa	Knock-out genotype (insertion size)	crRNA	Donor oligo	Confirmation PCR primers
MC1	*D. discoideum* AX2 *act5::mCherry*, *Hyg*	*mCherry* / aa9	*act5::mCherry* aa9 KO (random indel by NHEJ)	MC1	NA	MR101/ MR103
MC1_MR96	*D. discoideum* AX2 *act5::mCherry*, *Hyg*	*mCherry* / aa9	*act5::mCherry* aa9 KO (1bp)	MC1	MR96	MR101/ MR103
MC1_MR97	*D. discoideum* AX2 *act5::mCherry*, *Hyg*	*mCherry* / aa9	*act5::mCherry* aa9 KO (1bp)	MC1	MR97	MR101/ MR103
MC1_MR100	*D. discoideum* AX2 *act5::mCherry*, *Hyg*	*mCherry* / aa9	*act5::mCherry* aa9 KO (1bp)	MC1	MR100	MR101/ MR103
MC1_MR124	*D. discoideum* AX2 *act5::mCherry*, *Hyg*	*mCherry* / aa9	*act5::mCherry* aa9 KO (37bp)	MC1	MR124	MR101/ MR103
MC1_MR125	*D. discoideum* AX2 *act5::mCherry*, *Hyg*	*mCherry* / aa9	*act5::mCherry* aa9 KO (37bp)	MC1	MR125	MR101/ MR103
MC1_MR127	*D. discoideum* AX2 *act5::mCherry*, *Hyg*	*mCherry* / aa9	*act5::mCherry* aa9 KO (37bp)	MC1	MR127	MR101/ MR103
MC1_MR98	*D. discoideum* AX2 *act5::mCherry*, *Hyg*	*mCherry* / aa9	*act5::mCherry* aa9 KO (4bp)	MC1	MR98	MR101/ MR103
MC2_MR131	*D. discoideum* AX2 *act5::mCherry*, *Hyg*	*mCherry* / aa21	*act5::mCherry* aa21 KO (37bp)	MC2	MR131	MR101 /MR103
MC9_MR132	*D. discoideum* AX2 *act5::mCherry*, *Hyg*	*mCherry* / aa27	*act5::mCherry* aa27 KO (37bp)	MC9	MR132	MR101/ MR103
MC1_MR125_ NC4	*D. discoideum* NC4 *act5::mCherry*, *Hyg*	*mCherry* / aa9	*act5::mCherry* aa9 KO (37bp)	MC1	MR125	MR101/ MR103
MC29_MR223	*D. discoideum* AX2 *act5::mCherry*, *Hyg*	*pkaC* / aa9	*pkaC::pkaC* aa9 KO (37bp)	MC29	MR223	MR237/ MR238
MC30_MR224	*D. discoideum* AX2 *act5::mCherry*, *Hyg*	*pkaC* / aa22	*pkaC::pkaC* a22 KO (37bp)	MC30	MR224	MR237/ MR238
MC31_MR225	*D. discoideum* AX2 *act5::mCherry*, *Hyg*	*pkaC* / aa36	*pkaC::pkaC* a36 KO (37bp)	MC31	MR225	MR237/ MR238

[1] Transfection of *D. discoideum* AX2 with pDM1514 following Paschke *et al.* (2018).

Appendix Table S3. Analysis of knock-out strains using Nanopore sequencing[2]

Condition	Replicate	Description, sequence file with nanopore reads and alignment file[3]	Barcode	Overall coverage	% *mCherry* reads with correct edit (Alignment file to reference)[4]	% correct donor oligo integration (Alignment file to reference)[5]
Control 1	1	*D. discoideum* AX2<br>**Sequence file**: Control_1.fastq.gz<br>**Alignment file to reference**: Control_1_to_reference.bam	24	15.4x	NA	NA
Control 2	1	*D. discoideum* AX2 *act5::mCherry*<br>**Sequence file**: Control_2.fastq.gz<br>**Alignment file to reference**: Control_2_to_reference.bam	23	7.7x	100% correct (no edits, 8 reads) (Control_2_to_mcherry.bam)	NA
Control 3	1	Electroporated *D. discoideum* AX2 *act5::mCherry* cells, without RNP complex<br>**Sequence file**: Control_3_Rep1.fastq.gz<br>**Alignment file to reference**: Control_3_Rep1_to_reference.bam	21	16.2x	100% correct (no edits, 13 reads) (Control_3_Rep1_to_mcherry.bam)	NA
Control 3	2	Electroporated *D. discoideum* AX2 *act5::mCherry* cells, without RNP complex<br>**Sequence file**: Control_3_Rep2.fastq.gz<br>**Alignment file to reference**: Control_3_Rep2_to_reference.bam	22	18.0x	100% correct (no edits, 12 reads) (Control_3_Rep2_to_mcherry.bam)	NA
Insert	1	*D. discoideum* AX2 *act5::mCherry* transfected with RNP complex only (crRNA: MC1), Sanger sequencing shows a 16bp insertion in *mCherry* produced by NHEJ<br>**Sequence file**: Insert.fastq.gz<br>**Alignment file to reference**: Insert_to_reference.bam	16	15.1x	100% correct (16bp insertion, 13 reads) (Insert_to_mcherry.bam)	NA
Deletion	1	*D. discoideum* AX2 *act5::mCherry* transfected with RNP complex only (crRNA: MC1), Sanger sequencing shows a 4bp deletion in *mCherry* produced by NHEJ<br>**Sequence file**: Deletion.fastq.gz<br>**Alignment file to reference**: Deletion_to_reference.bam	17	25.8x	100% correct (4bp deletion, 21 reads) (Deletion_to_mcherry.bam)	NA
Donor oligo	1	*D. discoideum* AX2 *act5::mCherry* transfected with RNP complex (crRNA: MC1) and donor oligo (MR125). Sanger sequencing shows 37bp insert from donor oligo.<br>**Sequence file**: Donor_oligo_Rep1.fastq.gz<br>**Alignment file to reference**: Donor_oligo_Rep1_to_reference.bam	18	18.0x	100% correct (37bp insertion, 9 reads) (Donor_oligo_Rep1_to_mcherry.bam)	100% correct (5 reads) (Donor_oligo_Rep1_to_oligo.bam)
Donor oligo	2	*D. discoideum* AX2 *act5::mCherry* transfected with RNP complex (crRNA: MC1) and donor oligo (MR125). Sanger sequencing shows 37bp insert from donor oligo.<br>**Sequence file**: Donor_oligo_Rep2.fastq.gz<br>**Alignment file to reference**: Donor_oligo_Rep2_to_reference.bam	19	15.0x	100% correct (37bp insertion, 11 reads) (Donor_oligo_Rep2_to_mcherry.bam)	100% correct (9 reads) (Donor_oligo_Rep2_to_oligo.bam)
Donor oligo	3	*D. discoideum* AX2 *act5::mCherry* transfected with RNP complex (crRNA: MC1) and donor oligo (MR125). Sanger sequencing shows 37bp insert from donor oligo.<br>**Sequence file**: Donor_oligo_Rep3.fastq.gz<br>**Alignment file to reference**: Donor_oligo_Rep3_to_reference.bam	20	32.4x	100% correct (37bp insertion, 24 reads) (Donor_oligo_Rep3_to_mcherry.bam)	100% correct (10 reads) (Donor_oligo_Rep3_to_oligo.bam)

[2] Based on Nanopore sequencing, no indication of increased off-target SNPs or indels due to CRISPR-Cas9 editing could be detected.
[3] All Nanopore sequencing files, alignment files and alignment reference (our *D. discoideum* AX2 assembly) are available on Zenodo (https://doi.org/10.5281/zenodo.16919775)
[4] *mCherry* sequence flanking the cutting site was queried against all nanopore reads using BLAST 2.15.0+ to confirm that all *mCherry* reads were edited as expected.
[5] 37nt donor oligo (without homology arms) was queried against all nanopore reads using BLAST 2.15.0+ to confirm that all donor oligos were integrated at the expected *mCherry* target site.

Appendix Table S4. List of generated knock-in strains.

Identifier	Species / strain	Target gene / aa	Targeted locus tag[6] (GenBank assembly)	Knock-in genotype	crRNA	Donor PCR template	Donor PCR primers	Upstream junction PCR primers	Downstream junction PCR primer
MG060	*D. discoideum* AX2 *act5::mCherry, Hyg*	*mCherry / aa9*	NA	*act5::mNeonGreen-P2A-mCherry*	MC1	pMG005	AP1169/ AP1170	MR101/ MR113	MR112/ MR103
MG066	*D. discoideum* NC4	*act5 / aa1*	DDB_G0289663 (GCA_000004695.1)	*act5::mNeonGreen-act5*	MC5	pMG005	ME77/ MR78	MR101/ MR113	MR112/ MR102
MG067	*D. discoideum* AX2	*act5 / aa1*	DDB_G0289663 (GCA_000004695.1)	*act5:mNeonGreen-act5*	MC5	pMG005	ME77/ MR78	MR101/ MR113	MR112/ MR102
NA[7]	*D. discoideum* AX2	*H2Bv3 / aa4*	DDB_G0286509 (GCA_000004695.1)	*H2Bv3::mNeonGreen-H2Bv3*	MC3	pMG005	MR75/ ME76	MR85/ MR113	MR112/ MR86
NA	*D. discoideum* AX2	*H2Bv3 / aa155*	DDB_G0286509 (GCA_000004695.1)	*H2Bv3::H2Bv3-mNeonGreen*	MC4	pMG005	MR73/ MR74	MR89/ MR113	MR112/ MR88
MG077	*D. discoideum* AX2	*ecmA / aa27*	DDB_G0277853 (GCA_000004695.1)	*ecmA::mNeonGreen-P2A-ecmA*	MC10	pMG005	MR133/ MR134	MR146/ MR113	NA
MG075	*D. discoideum* AX2	*act5 / aa1*	DDB_G0289663 (GCA_000004695.1)	*act5::mNeonGreen-P2A-act5*	MC5	pMG005	MR77/ MR80	MR101/ MR113	MR112/ MR102
MG089	*D. discoideum* NC4	*act5 / aa1*	DDB_G0289663 (GCA_000004695.1)	*act5::mNeonGreen-P2A-act5*	MC5	pMG005	MR77/ MR80	MR101/ MR113	MR112/ MR102
MG137	*D. discoideum* AX2	*act5 / aa1, ecmA / aa27*	DDB_G0289663, DDB_G0277853 (GCA_000004695.1)	*act5::mCherry-P2A-act5, ecmA::mNeonGreen-P2A-ecmA*	MC5, MC10	pMG008, pMG005	MR169/ MR80, MR133/ MR134	MR101/ MR103, MR146/ MR113	MR32/ MR102
MG084	*P. violaceum* P6, S209	*act / aa12*	CYY_010281 (GCA_000277445.1)	*act::mNeonGreen-P2A-act*	MC13	pMG005	MR149/ MR150	MR193/ MR113	MR112/ MR157
MG100	*D. firmibasis* TNS-C-14	*act / aa1*	RB653_008742 (GCA_036169595.1)	*act::mNeonGreen-P2A-act*	MC12	pMG005	MR147/ MR148	MR153/ MR113	MR112/ MR156
MG130	*H. pallidum* PN500	*act* / aa8	PPL_06386 (GCA_000004825.1)	*act::mNeonGreen-P2A-act*	MC14	pMG005	MR151/ MR152	MR155/ MR113	MR112/ MR158
NA	*A. subglobosum* LB1	*act* / aa7	SAMD00019534_067160 (GCA_000787575.2)	*act::mNeonGreen-P2A-act*	MC16	pMG005	MR174/ MR175	NA	NA
MG116	*T. lacteum* 561	*act / aa13*	DLAC_02274 (GCA_001606155.1)	*act::mNeonGreen-P2A-act*	MC18	pMG005	MR172/ MR173	NA	MR112/ MR156
MG129	*D. purpureum* WS321	*act / aa12*	DICPUDRAFT_92254 (GCA_000190715.1)	*act::mNeonGreen-P2A-act*	MC13	pMG005	MR149/ MR150	MR154/ MR113	MR112/ MR157

[6] Correct integration of the donor DNA product at the targeted locus was confirmed by PCR with primers specific for the targeted locus and Sanger sequencing (File S7).
[7] No identifier associated, because *D. discoideum* AX2 *H2Bv3*::*H2Bv3-mNeonGreen*, *D. discoideum* AX2 *H2Bv3*::*mNeonGreen-H2Bv3* and *A. subglobosum act*::*mNeonGreen-P2A-act* strains could not be propagated due to deleterious growth effects.

Appendix Table S5. List of crRNAs.

Identifiers	Spacer sequence	Target: Strain / gene / aa
MC1	AAAAGGTGAAGAAGATAATA	*D. discoideum* / *mCherry* / aa9
MC2	TATGAGATTTAAAGTTCATA	*D. discoideum* / *mCherry* / a21
MC9	CATATGGAAGGTTCAGTTAA	*D. discoideum* / *mCherry* / aa27
MC28	TAATGGAGTTGATTTCGATA	*D. discoideum* / *mNeonGreen* / aa30
MC5	ATTATATATAAAAAAATGGA	*D. discoideum* / *act5* / aa1
MC3	TTCAAAATGGTATTCGTTAA	*D. discoideum* / *H2Bv3* / aa4
MC10	ACATGCTGTATTGGTTTGTA	*D. discoideum* / *ecmA* / aa27
MC4	ACTGAAAGCAAAAACTAAAT	*D. discoideum* / *H2Bv3* / aa155
MC12	AAAATACAATAAAAAATGGA	*D. firmibasis* / *act* / aa1
MC13	CAAGCTTTAGTTATCGATAA	*D. purpureum* / *act* / aa12
MC14	AGGTGAAGACGTTCAAGCTT	*H. pallidum* / *act* / aa8
MC16	AGGAGAAGACGTTCAAGCTT	*A. subglosum* / *act5* / aa7
MC18	TGATGGGAATACAGCTCTTG	*T. lacteum* / *act* / aa13
MC13	CAAGCTTTAGTTATCGATAA	*P. violaceum* / *act* / aa12
MC29	TCAAATAATAATAGCTCAAG	*D. discoideum* / *pkaC* / aa9
MC30	TGCTATATACATTAACTTTA	*D. discoideum* / *pkaC* / aa22
MC31	AATTCAACAACATACACATA	*D. discoideum* / *pkaC* / aa36

Appendix Table S6. List of oligos (homology arms in blue).

Identifier	Sequence	Description
MR96	AAGGTGAAGAAGATTAATATGGCAATTAT	Donor oligo. Target: *mCherry* aa9. Insertion: 1bp. Homology arms: 14bp.
MR97	AAAAATGGTTTCAAAAGGTGAAGAAGATTAATAT GGCAATTATTAAAGAATTTATGA	Donor oligo. Target: *mCherry* aa9. Insertion: 1bp. Homology arms: 28bp.
MR100	AAAATAAATTATATATAAAAAAGATCCAAAAAAT GGTTTCAAAAGGTGAAGAAGATTAATATGGCAAT TATTAAAGAATTTATGAGATTTAAAGTTCATATG GAAGGTTCAGT	Donor oligo. Target: *mCherry* aa9. Insertion: 1bp. Homology arms: 56bp.
MR124	AAGGTGAAGAAGATTACCCCTACGACGTGCCAGA TTACGCCTCTCTGTAATAATATGGCAATTAT	Donor oligo. Target: *mCherry* aa9. Insertion: 37bp. Homology arms: 14bp.
MR125	AAAAATGGTTTCAAAAGGTGAAGAAGATTACCCC TACGACGTGCCAGATTACGCCTCTCTGTAATAAT ATGGCAATTATTAAAGAATTTATGA	Donor oligo. Target: *mCherry* aa9. Insertion: 37bp. Homology arms: 28bp.
MR127	AAAATAAATTATATATAAAAAAGATCCAAAAAAT GGTTTCAAAAGGTGAAGAAGATTACCCCTACGAC GTGCCAGATTACGCCTCTCTGTAATAATATGGCA ATTATTAAAGAATTTATGAGATTTAAAGTTCATA TGGAAGGTTCAGT	Donor oligo. Target: *mCherry* aa9. Insertion: 37bp. Homology arms: 56bp.
MR98	AAAAATGGTTTCAAAAGGTGAAGAAGATTAATAA TATGGCAATTATTAAAGAATTTATGA	Donor oligo. Target: *mCherry* aa9. Insertion: 4bp. Homology arms: 28bp.
MR131	TATTAAAGAATTTATGAGATTTAAAGTTTACCCC TACGACGTGCCAGATTACGCCTCTCTGTAATAAT ATGGAAGGTTCAGTTAATGGTCATGA	Donor oligo. Target: *mCherry* aa21. Insertion: 37bp. Homology arms: 28bp.
MR132	GAGATTTAAAGTTCATATGGAAGGTTCATACCCC TACGACGTGCCAGATTACGCCTCTCTGTAATGTT AATGGTCATGAATTTGAAATTGAAG	Donor oligo. Target: *mCherry* aa27. Insertion: 37bp. Homology arms: 28bp.
AP1169	GATCCAAAAAATGGTTTCAAAAGGTGAAGAAGAT GTATCTAAGGGAGAAGAAG	Forward primer for *act5*::*mNeonGreen-P2A-mCherry* knock-in. Template: pMG005.
AP1170	CTTTAAATCTCATAAATTCTTTAATAATTGCCAT ATTAGAACTAGATCCACTAGGTC	Reverse primer for *act5*::*mNeonGreen-P2A-mCherry* knock-in. Template: pMG005.
MR73	CTGTCAACAAGTACAATCCAACTGAAAGCAAAAA CGACTACAAAGATCACGATG	Forward primer for *H2Bv3*::*mNeonGreen-H2Bv3* knock-in. Template: pMG005.
MR74	TTTTTAAGGAATATAGTTCATTTGGAACCAATTT ACTTATATAATTCATCCATTCC	Reverse primer for *H2Bv3*::*mNeonGreen-H2Bv3* knock-in. Template: pMG005.
MR75	AATAGTCAATTAATATAATTCAAAATGGTATTCG TATCTAAGGGAGAAGAAG	Forward primer for *H2Bv3*::*H2Bv3-mNeonGreen* knock-in. Template: pMG005.
ME76	TGAGTTGAACCTTTGGTTGCTTTCTTTTGACCTT TAACACCAGAACCACCAGCATAA	Reverse primer for *H2Bv3*::*H2Bv3-mNeonGreen* knock-in. Template: pMG005.
ME77	ATAAAAACTTAAAATAAATTATATATAAAAAAAT GGTATCTAAGGGAGAAGAAG	Forward primer for *act5*::*mNeonGreen-act5* and *act5*::*mNeonGreen-P2A-act5* knock-in. Template: pMG005.
MR169	ATAAAAACTTAAAATAAATTATATATAAAAAAAT GGTTTCAAAAGGTGAAGAAG	Forward primer for *act5*::*mCherry-P2A-act5* knock-in. Template: pMG008
MR80	TTATCGATAACTAAAGCTTGAACATCTTCACCGT CAGAACTAGATCCACTAGGTC	Reverse primer for *act5*::*mNeonGreen-P2A-act5* and *act5*::*mCherry-P2A-act5* knock-in. Template: pMG005 and pMG008, respectively.
MR78	TTATCGATAACTAAAGCTTGAACATCTTCACCGT CACCAGAACCACCAGCATAA	Reverse primer for *act5*::*mNeonGreen-act5* knock-in. Template: pMG005.
MR133	TAATATTTAATAGCGGAAACTGAAACCATAGTAT CTAAGGGAGAAGAAG	Forward primer for *ecmA*::*mNeonGreen-P2A-ecmA* knock-in. Template: pMG005.
MR134	ATCATCACAATCACATGCTGTATTGGTTTGAGAA CTAGATCCACTAGGTC	Reverse primer for *ecmA*::*mNeonGreen-P2A-ecmA* knock-in. Template: pMG005.
MR147	ATTTCAAACTATAATATATAAAATACAATAAAAA ATGGTATCTAAGGGAGAAGAAG	Forward primer for *D. firmibasis act*::*mNeonGreen-P2A-act* knock-in. Template: pMG005.
MR148	TATCGATAACTAAAGCTTGAACATCTTCACCTTC AGAACTAGATCCACTAGGTC	Reverse primer for *D. firmibasis act*::*mNeonGreen-P2A-act* knock-in. Template: pMG005.
MR149	AGATGGAAGGTGAAGATGTTCAAGCTTTAGTTAT CGTATCTAAGGGAGAAGAAG	Forward primer for *D. purpureum act*::*mNeonGreen-P2A-act* knock-in. Template: pMG005.
MR150	GCAAAACCGGCTTTGCACATACCTGAACCGTTAT CAGAACTAGATCCACTAGGTC	Reverse primer for *D. purpureum act*::*mNeonGreen-P2A-act* knock-in. Template: pMG005.
MR151	ATAATAAATAAACAATGGAAGGTGAAGACGTTCA AGTATCTAAGGGAGAAGAAG	Forward primer for *H. pallidum act*::*mNeonGreen-P2A-act* knock-in. Template: pMG005.
MR152	CTAGATGATCAACATACGTTATCAATTACCAAAG CAGAACTAGATCCACTAGGTC	Reverse primer for *H. pallidum act*::*mNeonGreen-P2A-act* knock-in. Template: pMG005.
MR172	TGTGTAAAGCCGGTTTTGCTGGTGATGATGCCCC AGTATCTAAGGGAGAAGAAG	Forward primer for *T. lacteum act*::*mNeonGreen-P2A-act* knock-in. Template: pMG005.

MR173	CTTGGACGACCGACAATTGATGGGAATACAGCTC TAGAACTAGATCCACTAGGTC	Reverse primer for *T. lacteum act*::*mNeonGreen-P2A-act* knock-in. Template: pMG005.
MR174	TAATAATAATAATAATGGAAGGAGAAGACGTTCA AGTATCTAAGGGAGAAGAAG	Forward primer for *A. subglobosum act*::*mNeonGreen-P2A-act* knock-in. Template: pMG005.
MR175	TTGCACATACCGGAACCGTTGTCAATGACCAAAG CAGAACTAGATCCACTAGGTC	Reverse primer for *A. subglobosum act*::*mNeonGreen-P2A-act* knock-in. Template: pMG005.
MR63	GTTCCTTCCACCGGATACTTG	Reverse primer confirmation PCR. Target: downstream of *act5*
MR129	TACCCCTACGACGTGCCA	Forward primer confirmation PCR. Target: *HA-tag*
MR128	TTACAGAGAGGCGTAATCTGG	Reverse primer confirmation PCR. Target: *HA-tag*
MR103	TCCCATGCAAATGGTAATGGAC	Reverse primer confirmation PCR. Target: *mCherry*
MR32	ATGGGTTGGGAAGCATCATCA	Forward primer confirmation PCR. Target: *mCherry*
MR245	GATAATATGGCAATTATTAAAGAATTTATGAG	Forward primer confirmation PCR. Target: *mCherry*
MR112	CCAAGCCAATGGCAGCAAAT	Forward primer confirmation PCR. Target: *mNeonGreen*
MR113	AGCGGCTTGAAATGGACTCA	Reverse primer confirmation PCR. Target: *mNeonGreen*
MR114	TATGGCCTCTCTTCCTGCCA	Forward primer confirmation PCR. Target: *mNeonGreen*
MR101	CACCAGGTATATTACCAGATGGC	Forward primer confirmation PCR. Target: upstream of *act5*
MR102	TCTGGAGCAACACGGAGTTC	Reverse primer confirmation PCR. Target: downstream of *act5*
MR89	TCATTCTCACTGGTGAGTTAGC	Forward primer confirmation PCR. Target: *H2Bv3*
MR86	GGTGGTTGAAGCGGTTTTCTC	Reverse primer confirmation PCR. Target: *H2Bv3*
MR88	CATCCCAATCGATGGCATTCA	Reverse primer confirmation PCR. Target: downstream of *H2Bv3*
MR146	TGTAAACTCGTGAGGGGGTTGG	Forward primer confirmation PCR. Target: upstream of *ecmA*
MR153	GCCAACCGATATTGATAACGACC	Forward primer confirmation PCR. Target: upstream of *D. firmibasis act*.
MR154	CAGGTTTTGAAAGTTGGGAAGCC	Forward primer confirmation PCR. Target: upstream of *D. purpureum act*.
MR155	TGAGCAATCGATTGGAGTTGG	Forward primer confirmation PCR. Target: upstream of *H. pallidum act*.
MR156	GAGTCTTTTTGACCCATACCGACC	Reverse primer confirmation PCR. Target: *D. firmibasis act*.
MR157	CTCTTTGATTGGGCTTCATCACC	Forward primer confirmation PCR. Target: *D. purpureum act*.
MR193	GGAAGGTGAAGACGTTCAAGC	Forward primer confirmation PCR. Target: *P. violaceum act*.
MR158	GTCTTGGACGACCGACGATC	Reverse primer confirmation PCR. Target *H. pallidum act*.
MR217	TTTCGGTTCTATTAATGGAGTTGATTTCGAACAG AAACTCATCTCGGAGGAGGACCTGTAATAATGAT ATGGTTGGACAAGGAACTGGTAATC	Donor oligo. Target: *mNeonGreen* aa30. Insertion: *Myc-tag* 37bp. Homology arms: 28bp.
MR218	GAGATTTAAAGTTCATATGGAAGGTTCAGACTAC AAAGATCACGATGGAGATTATAAGGATTAATGTT AATGGTCATGAATTTGAAATTGAAG	Donor oligo. Target: *mCherry* aa27. Insertion: *FLAG -tag* 37bp. Homology arms: 28bp.
MR219	GAAACTCATCTCGGAGGAGGAC	Forward primer confirmation PCR. Target: *Myc-tag*
MR220	GTCCTCCTCCGAGATGAGTTTC	Reverse primer confirmation PCR. Target: *Myc-tag*
MR221	CAAAGATCACGATGGAGATTATAAGG	Forward primer confirmation PCR. Target: *FLAG-tag*
MR222	CCTTATAATCTCCATCGTGATCTTTG	Reverse primer confirmation PCR. Target: *FLAG-tag*
MR223	CATGAGTAACTCAAATAATAATAGCTCATACCCC TACGACGTGCCAGATTACGCCTCTCTGTAATAGT GGTAATCATAATAGTACAACAATAA	Donor oligo. Target: *pkaC* aa9. Insertion: 37bp. Homology arms: 28bp.
MR224	TAATAGTACAACAATAAATAACCCTAAATACCCC TACGACGTGCCAGATTACGCCTCTCTGTAATGTT AATGTATATAGCAATATACCAAATT	Donor oligo. Target: *pkaC* aa22. Insertion: 37bp. Homology arms: 28bp.
MR225	CAATATACCAAATTCAACAACATACACATACCCC TACGACGTGCCAGATTACGCCTCTCTGTAATTAT GGCAGCGGAGGAGGTGGAACACTAA	Donor oligo. Target: *pkaC* aa36. Insertion: 37bp. Homology arms: 28bp.
MR230	CGGGTTCACCTTCAACTTTCCTTGCGACTACCCC TACGACGTGCCAGATTACGCCTCTCTGTAATTAT AGGCGAAGAGTCAAACGAATAGAAA	Donor oligo. Target: *tgrB1* aa106. Insertion: 37bp. Homology arms: 28bp.

MR232	ATATTTAATAGCGGAAACTGAAACCATATACCCC TACGACGTGCCAGATTACGCCTCTCTGTAATCAA ACCAATACAGCATGTGATTGTGATG	Donor oligo. Target: *ecmA* aa27. Insertion: 37bp. Homology arms: 28bp.
MR233	TTGTGATGATGATTGTGATGATGGAAATTACCCC TACGACGTGCCAGATTACGCCTCTCTGTAATAGA TGGTCAACAGATATGTGTTCTGCAG	Donor oligo. Target: *ecmA* aa43. Insertion: 37bp. Homology arms: 28bp.
MR234	AAATAGATGGTCAACAGATATGTGTTCTTACCCC TACGACGTGCCAGATTACGCCTCTCTGTAATGCA GGTTTACTTTTTGGAAAATATTGCA	Donor oligo. Target: *ecmA* aa51. Insertion: 37bp. Homology arms: 28bp.
MR237	ACATGAGTAACTCAAATAATAATAGCTC	Forward primer confirmation PCR. Target: *pkaC*
MR238	GTATTATTTCCACTTAGTGTTCCACC	Reverse primer confirmation PCR. Target: *pkaC*
MR241	GTAAATTTTTATTTGTTAAATCAAGTTGCTC	Forward primer confirmation PCR. Target: *tgrB1*
MR242	CCTGGATTCAAGTAAGTAACGAAC	Reverse primer confirmation PCR. Target: *tgrB1*
MR243	AGCGGAAACTGAAACCATA	Forward primer confirmation PCR. Target: *ecmA*
MR244	CACAACAAATTTGAGTATGAGTGC	Reverse primer confirmation PCR. Target: *ecmA*
18S_D142 F	TGGATAACCGCAGTAAACGGG	1st round of PCR for 18S sequencing[8]
18SR_B	TGATCCTTCTGCAGGTTCAC	1st round of PCR for 18S sequencing[8]
18S_D307 F	GTTTGGCCTACCATGGTTGTAA	2nd round of PCR for 18S sequencing[8]
18S_D862 R	CACCTCTCGCCCCAATATGA	2nd round of PCR for 18S sequencing[8]
MR44	GATCCAAAAAATGGGAGTAGCTGATTTAATAAAA AAATTTGAATCAATATCTAAAGAAGA	Non-homologous oligo (Appendix Figure S10)

[8] (Baldauf *et al*, 2018)

Appendix Text S1. Vector sequences.

>pMG005 (kozak like-Start Codon-SV40-NLS-Linker3-nuleoplasmin NLS- 3xFLAG-tag-Codon optimized mNeonGreen-HA-tag-Linker1-P2A-Linker2B-Stop Codon-act8T)

CTAAATTGTAAGCGTTAATATTTTGTTAAAATTCGCGTTAAATTTTTGTTAAATCAGCTCATTTTTTAACCAATAGGCCGAAATCGGCAA
AATCCCTTATAAATCAAAAGAATAGACCGAGATAGGGTTGAGTGGCCGCTACAGGGCGCTCCCATTCGCCATTCAGGCTGCGCAACTGTT
GGGAAGGGCGTTTCGGTGCGGGCCTCTTCGCTATTACGCCAGCTGGCGAAAGGGGGATGTGCTGCAAGGCGATTAAGTTGGGTAACGCCA
GGGTTTTCCCAGTCACGACGTTGTAAAACGACGGCCAGTGAGCGCGACGTAATACGACTCACTATAGGGCGAATTGGCGGAAGGCCGTCA
AGGCCGCATAAAAAATGGTGAGTTCATTAAGACCACCAAAGAAAAAGAGAAAGGTTTGTCCAGGTGATAGATGGAGTAGTACAGGAGGTG
GAAGAAGTAGAACTTCAAAGAGACCTGCCGCTACAAAAAAGGCCGGACAAGCAAAGAAGAAAAAGGACTACAAAGATCACGATGGAGATT
ATAAGGATCACGATATAGATTACAAAGATGATGATGATAAAGTATCTAAGGGAGAAGAAGATAATATGGCCTCTCTTCCTGCCACCCATG
AATTACACATTTTCGGTTCTATTAATGGAGTTGATTTCGATATGGTTGGACAAGGAACTGGTAATCCAAATGATGGTTATGAGGAATTAA
ATCTTAAAAGTACAAAGGGAGATCTTCAATTTAGTCCATGGATCTTAGTACCTCACATCGGATACGGTTTTCACCAATACTTGCCATACC
CAGATGGTATGAGTCCATTTCAAGCCGCTATGGTAGATGGAAGTGGTTACCAAGTACACCGTACAATGCAATTTGAAGATGGAGCTAGTC
TTACAGTTAATTACCGTTATACATACGAGGGATCTCATATCAAAGGTGAGGCCCAAGTAAAGGGAACCGGTTTCCCTGCAGATGGACCAG
TAATGACTAATAGTTTAACTGCCGCCGATTGGTGTAGATCAAAAAAGACATATCCAAATGATAAGACTATAATATCAACATTCAAATGGT
CTTACACTACTGGAAATGGTAAGAGATATCGTTCTACCGCCAGAACAACTTACACATTTGCCAAGCCAATGGCAGCAAATTACTTAAAGA
ATCAACCTATGTACGTTTTTCGTAAAACAGAGTTGAAGCACTCAAAGACCGAGTTGAATTTTAAAGAGTGGCAAAAGGCATTTACAGATG
TTATGGGAATGGATGAATTATATAAGTACCCATACGATGTACCTGATTATGCTGGTGGTTCTGGTGCTACAAATTTCTCTTTGTTAAAGC
AAGCTGGAGATGTTGAAGAAAATCCTGGACCTTCTAGTGGACCTAGTGGATCTAGTTCTTGAATTATTTAATAAATAATAAAAAAACAAA
TTGTTGTAATAATCTAATATTTTCTTTTTTTTTTAATTTTTTTTTTTTTAAATCTTAATAATTATTAAGTTATTTTAATTTTTTTTTTTT
TTTTTTTTTTTTTTTTTTTTTTTTTCTATCAAAAAAATCAAATATATTTAAAAAATTTATTATTTACAGATACATTTTGAATGGTGAAGA
TAAATATATGCATTAGATGTAAAACAGCCAAAGAGTATGAAAATCAAAAAGATACTGGGCCTCATGGGCCTTCCGCTCACTGCCCGCTTT
CCAGTCGGGAAACCTGTCGTGCCAGCTGCATTAACATGGTCATAGCTGTTTCCTTGCGTATTGGGCGCTCTCCGCTTCCTCGCTCACTGA
CTCGCTGCGCTCGGTCGTTCGGGTAAAGCCTGGGGTGCCTAATGAGCAAAAGGCCAGCAAAAGGCCAGGAACCGTAAAAAGGCCGCGTTG
CTGGCGTTTTTCCATAGGCTCCGCCCCCCTGACGAGCATCACAAAAATCGACGCTCAAGTCAGAGGTGGCGAAACCCGACAGGACTATAA
AGATACCAGGCGTTTCCCCCTGGAAGCTCCCTCGTGCGCTCTCCTGTTCCGACCCTGCCGCTTACCGGATACCTGTCCGCCTTTCTCCCT
TCGGGAAGCGTGGCGCTTTCTCATAGCTCACGCTGTAGGTATCTCAGTTCGGTGTAGGTCGTTCGCTCCAAGCTGGGCTGTGTGCACGAA
CCCCCCGTTCAGCCCGACCGCTGCGCCTTATCCGGTAACTATCGTCTTGAGTCCAACCCGGTAAGACACGACTTATCGCCACTGGCAGCA
GCCACTGGTAACAGGATTAGCAGAGCGAGGTATGTAGGCGGTGCTACAGAGTTCTTGAAGTGGTGGCCTAACTACGGCTACACTAGAAGA
ACAGTATTTGGTATCTGCGCTCTGCTGAAGCCAGTTACCTTCGGAAAAAGAGTTGGTAGCTCTTGATCCGGCAAACAAACCACCGCTGGT
AGCGGTGGTTTTTTTGTTTGCAAGCAGCAGATTACGCGCAGAAAAAAAGGATCTCAAGAAGATCCTTTGATCTTTTCTACGGGGTCTGAC
GCTCAGTGGAACGAAAACTCACGTTAAGGGATTTTGGTCATGAGATTATCAAAAAGGATCTTCACCTAGATCCTTTTAAATTAAAAATGA
AGTTTTAAATCAATCTAAAGTATATATGAGTAAACTTGGTCTGACAGTTACCAATGCTTAATCAGTGAGGCACCTATCTCAGCGATCTGT
CTATTTCGTTCATCCATAGTTGCCTGACTCCCCGTCGTGTAGATAACTACGATACGGGAGGGCTTACCATCTGGCCCCAGTGCTGCAATG
ATACCGCGAGAACCACGCTCACCGGCTCCAGATTTATCAGCAATAAACCAGCCAGCCGGAAGGGCCGAGCGCAGAAGTGGTCCTGCAACT
TTATCCGCCTCCATCCAGTCTATTAATTGTTGCCGGGAAGCTAGAGTAAGTAGTTCGCCAGTTAATAGTTTGCGCAACGTTGTTGCCATT
GCTACAGGCATCGTGGTGTCACGCTCGTCGTTTGGTATGGCTTCATTCAGCTCCGGTTCCCAACGATCAAGGCGAGTTACATGATCCCCC
ATGTTGTGCAAAAAAGCGGTTAGCTCCTTCGGTCCTCCGATCGTTGTCAGAAGTAAGTTGGCCGCAGTGTTATCACTCATGGTTATGGCA
GCACTGCATAATTCTCTTACTGTCATGCCATCCGTAAGATGCTTTTCTGTGACTGGTGAGTACTCAACCAAGTCATTCTGAGAATAGTGT
ATGCGGCGACCGAGTTGCTCTTGCCCGGCGTCAATACGGGATAATACCGCGCCACATAGCAGAACTTTAAAAGTGCTCATCATTGGAAAA
CGTTCTTCGGGGCGAAAACTCTCAAGGATCTTACCGCTGTTGAGATCCAGTTCGATGTAACCCACTCGTGCACCCAACTGATCTTCAGCA
TCTTTTACTTTCACCAGCGTTTCTGGGTGAGCAAAAACAGGAAGGCAAAATGCCGCAAAAAAGGGAATAAGGGCGACACGGAAATGTTGA
ATACTCATACTCTTCCTTTTTCAATATTATTGAAGCATTTATCAGGGTTATTGTCTCATGAGCGGATACATATTTGAATGTATTTAGAAA
AATAAACAAATAGGGGTTCCGCGCACATTTCCCCGAAAAGTGCCAC

>pMG008 (kozak like-Start Codon-SV40-NLS-Linker3-nuleoplasmin NLS- 3xFLAG-tag-Codon optimized mCherry-HA-tag-Linker1-P2A-Linker2B-Stop Codon-act8T)

CTAAATTGTAAGCGTTAATATTTTGTTAAAATTCGCGTTAAATTTTTGTTAAATCAGCTCATTTTTTAACCAATAGGCCGAAATCGGCAA
AATCCCTTATAAATCAAAAGAATAGACCGAGATAGGGTTGAGTGGCCGCTACAGGGCGCTCCCATTCGCCATTCAGGCTGCGCAACTGTT
GGGAAGGGCGTTTCGGTGCGGGCCTCTTCGCTATTACGCCAGCTGGCGAAAGGGGGATGTGCTGCAAGGCGATTAAGTTGGGTAACGCCA
GGGTTTTCCCAGTCACGACGTTGTAAAACGACGGCCAGTGAGCGCGACGTAATACGACTCACTATAGGGCGAATTGGCGGAAGGCCGTCA
AGGCCGCATAAAAAATGGTGAGTTCATTAAGACCACCAAAGAAAAAGAGAAAGGTTTGTCCAGGTGATAGATGGAGTAGTACAGGAGGTG
GAAGAAGTAGAACTTCAAAGAGACCTGCCGCTACAAAAAGGCCGGACAAGCAAAGAAGAAAAAGGACTACAAAGATCACGATGGAGATT
ATAAGGATCACGATATAGATTACAAAGATGATGATGATAAAGTTTCAAAAGGTGAAGAAGATAATATGGCAATTATTAAAGAATTTATGA
GATTTAAAGTTCATATGGAAGGTTCAGTTAATGGTCATGAATTTGAAATTGAAGGTGAAGGTGAAGGTAGACCATATGAAGGTACACAAA
CAGCAAAATTAAAAGTTACAAAAGGTGGTCCATTACCATTTGCATGGGATATTTTATCACCACAATTTATGTATGGTTCAAAAGCATATG
TTAAACATCCAGCAGATATTCCAGATTATTTAAAATTATCATTTCCAGAAGGTTTTAAATGGGAAAGAGTTATGAATTTTGAAGATGGTG
GTGTTGTTACAGTTACACAAGATTCATCATTACAAGATGGTGAATTTATTTATAAAGTTAAATTAAGAGGTACAAATTTTCCATCAGATG
GTCCAGTTATGCAAAAGAAAACAATGGGTTGGGAAGCATCATCAGAAAGAATGTATCCAGAAGATGGTGCATTAAAAGGTGAAATTAAAC
AAAGATTAAAATTAAAAGATGGTGGTCATTATGATGCAGAAGTTAAAACAACATATAAAGCAAAAAAACCAGTTCAATTACCAGGTGCAT
ATAATGTTAATATTAAATTAGATATTACATCACATAATGAAGATTATACAATTGTTGAACAATATGAAAGAGCAGAAGGTAGACATTCAA
CAGGTGGTATGGATGAATTATATAAATACCCATACGATGTACCTGATTATGCTGGTGGTTCTGGTGCTACAAATTTCTCTTTGTTAAAGC
AAGCTGGAGATGTTGAAGAAAATCCTGGACCTTCTAGTGGACCTAGTGGATCTAGTTCTTGAATTATTTAATAAATAATAAAAAAACAAA
TTGTTGTAATAATCTAATATTTTCTTTTTTTTTTAATTTTTTTTTTTTAAATCTTAATAATTATTAAGTTATTTTAATTTTTTTTTTTTT
TTTTTTTTTTTTTTTTTTTTTTTTCTATCAAAAAAATCAAATATATTTAAAAAATTTATTATTTACAGATACATTTTGAATGGTGAAGA
TAAATATATGCATTAGATGTAAAACAGCCAAAGAGTATGAAAATCAAAAAGATACTGGGCCTCATGGGCCTTCCGCTCACTGCCCGCTTT
CCAGTCGGGAAACCTGTCGTGCCAGCTGCATTAACATGGTCATAGCTGTTTCCTTGCGTATTGGGCGCTCTCCGCTTCCTCGCTCACTGA
CTCGCTGCGCTCGGTCGTTCGGGTAAAGCCTGGGGTGCCTAATGAGCAAAAGGCCAGCAAAAGGCCAGGAACCGTAAAAAGGCCGCGTTG
CTGGCGTTTTTCCATAGGCTCCGCCCCCCTGACGAGCATCACAAAAATCGACGCTCAAGTCAGAGGTGGCGAAACCCGACAGGACTATAA
AGATACCAGGCGTTTCCCCCTGGAAGCTCCCTCGTGCGCTCTCCTGTTCCGACCCTGCCGCTTACCGGATACCTGTCCGCCTTTCTCCCT
TCGGGAAGCGTGGCGCTTTCTCATAGCTCACGCTGTAGGTATCTCAGTTCGGTGTAGGTCGTTCGCTCCAAGCTGGGCTGTGTGCACGAA
CCCCCCGTTCAGCCCGACCGCTGCGCCTTATCCGGTAACTATCGTCTTGAGTCCAACCCGGTAAGACACGACTTATCGCCACTGGCAGCA
GCCACTGGTAACAGGATTAGCAGAGCGAGGTATGTAGGCGGTGCTACAGAGTTCTTGAAGTGGTGGCCTAACTACGGCTACACTAGAAGA
ACAGTATTTGGTATCTGCGCTCTGCTGAAGCCAGTTACCTTCGGAAAAAGAGTTGGTAGCTCTTGATCCGGCAAACAAACCACCGCTGGT
AGCGGTGGTTTTTTTGTTTGCAAGCAGCAGATTACGCGCAGAAAAAAAGGATCTCAAGAAGATCCTTTGATCTTTTCTACGGGGTCTGAC
GCTCAGTGGAACGAAAACTCACGTTAAGGGATTTTGGTCATGAGATTATCAAAAAGGATCTTCACCTAGATCCTTTTAAATTAAAAATGA
AGTTTTAAATCAATCTAAAGTATATATGAGTAAACTTGGTCTGACAGTTACCAATGCTTAATCAGTGAGGCACCTATCTCAGCGATCTGT
CTATTTCGTTCATCCATAGTTGCCTGACTCCCCGTCGTGTAGATAACTACGATACGGGAGGGCTTACCATCTGGCCCCAGTGCTGCAATG
ATACCGCGAGAACCACGCTCACCGGCTCCAGATTTATCAGCAATAAACCAGCCAGCCGGAAGGGCCGAGCGCAGAAGTGGTCCTGCAACT
TTATCCGCCTCCATCCAGTCTATTAATTGTTGCCGGGAAGCTAGAGTAAGTAGTTCGCCAGTTAATAGTTTGCGCAACGTTGTTGCCATT
GCTACAGGCATCGTGGTGTCACGCTCGTCGTTTGGTATGGCTTCATTCAGCTCCGGTTCCCAACGATCAAGGCGAGTTACATGATCCCCC
ATGTTGTGCAAAAAAGCGGTTAGCTCCTTCGGTCCTCCGATCGTTGTCAGAAGTAAGTTGGCCGCAGTGTTATCACTCATGGTTATGGCA
GCACTGCATAATTCTCTTACTGTCATGCCATCCGTAAGATGCTTTTCTGTGACTGGTGAGTACTCAACCAAGTCATTCTGAGAATAGTGT
ATGCGGCGACCGAGTTGCTCTTGCCCGGCGTCAATACGGGATAATACCGCGCCACATAGCAGAACTTTAAAAGTGCTCATCATTGGAAAA
CGTTCTTCGGGGCGAAAACTCTCAAGGATCTTACCGCTGTTGAGATCCAGTTCGATGTAACCCACTCGTGCACCCAACTGATCTTCAGCA
TCTTTTACTTTCACCAGCGTTTCTGGGTGAGCAAAAACAGGAAGGCAAAATGCCGCAAAAAAGGGAATAAGGGCGACACGGAAATGTTGA
ATACTCATACTCTTCCTTTTTCAATATTATTGAAGCATTTATCAGGGTTATTGTCTCATGAGCGGATACATATTTGAATGTATTTAGAAA
AATAAACAAATAGGGGTTCCGCGCACATTTCCCCGAAAAGTGCCAC

Appendix Text S2. Detailed protocol.

Selection-free CRISPR-Cas9 editing protocol for distant *Dictyostelid* species

Table of contents

	Page
**Graphic protocol**	39
**List of materials**	40
**Buffers and reactions**	42
**Part 1. CRISPR-Cas9 editing protocol with RNP complex in *D. discoideum***	46
**Step 1**. Identifying good CRISPR-Cas9 targets	46
**Step 2**. Designing donor oligos and donor PCR products	47
**Step 3**. Culturing axenic and non-axenic strains of *D. discoideum*	51
**Step 4**. Transfecting cells with RNP complex by electroporation	52
**Step 5**. Flow cytometry analysis	55
**Step 6**. Isolation of gene-edited cells using cell sorting	56
**Step 7**. Propagation of positive clones	59
**Part 2. Notes for CRISPR-Cas9 editing in other *Dictyostelid* species**	60

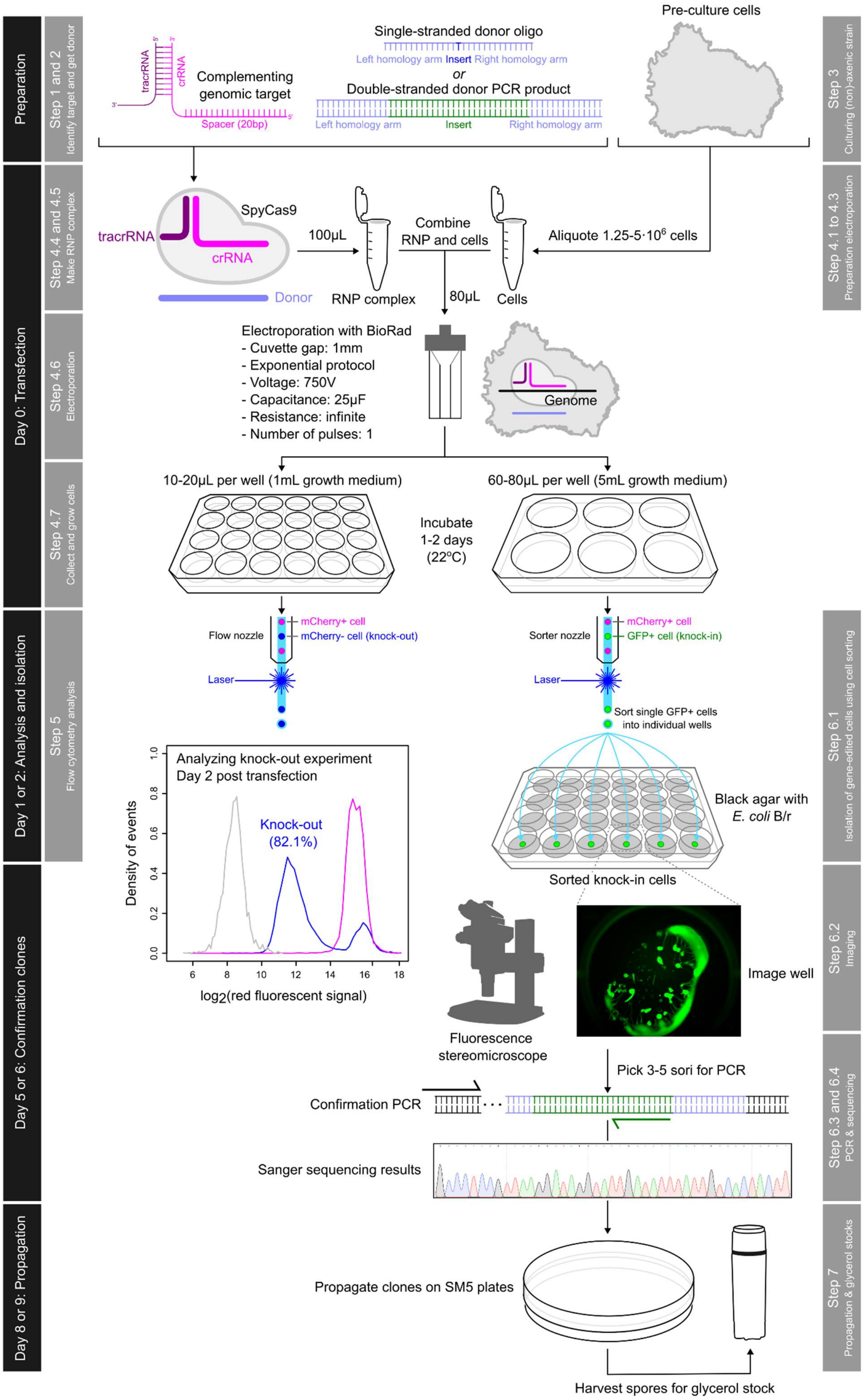
Preparation
Step 1 and 2
Identify target and get donor
tracrRNA
crRNA
Complementing genomic target
Spacer (20bp)
Single-stranded donor oligo
Left homology arm Insert Right homology arm
or
Double-stranded donor PCR product
Left homology arm
Insert
Right homology arm
Pre-culture cells
Step 3
Culturing (non)-axenic strain
Day 0: Transfection
Step 4.4 and 4.5
Make RNP complex
SpyCas9
tracrRNA
crRNA
Donor
100µL
RNP complex
Combine RNP and cells
80µL
Cells
Aliquote 1.25-5·10^6 cells
Step 4.1 to 4.3
Preparation electroporation
Step 4.6
Electroporation
Electroporation with BioRad
- Cuvette gap: 1mm
- Exponential protocol
- Voltage: 750V
- Capacitance: 25µF
- Resistance: infinite
- Number of pulses: 1
Genome
Step 4.7
Collect and grow cells
10-20µL per well (1mL growth medium)
Incubate 1-2 days (22°C)
60-80µL per well (5mL growth medium)
Day 1 or 2: Analysis and isolation
Step 5
Flow cytometry analysis
Flow nozzle
mCherry+ cell
mCherry- cell (knock-out)
Laser
Sorter nozzle
mCherry+ cell
GFP+ cell (knock-in)
Laser
Sort single GFP+ cells into individual wells
Step 6.1
Isolation of gene-edited cells using cell sorting
Analyzing knock-out experiment
Day 2 post transfection
Knock-out (82.1%)
Density of events
log2(red fluorescent signal)
Black agar with E. coli B/r
Sorted knock-in cells
Day 5 or 6: Confirmation clones
Step 6.2
Imaging
Fluorescence stereomicroscope
Image well
Pick 3-5 sori for PCR
Confirmation PCR
Step 6.3 and 6.4
PCR & sequencing
Sanger sequencing results
Day 8 or 9: Propagation
Step 7
Propagation & glycerol stocks
Propagate clones on SM5 plates
Harvest spores for glycerol stock

List of materials

Material	Provider	Catalogue number/Reference
**Software**		
Snapgene		https://www.snapgene.com
Cas designer		http://www.rgenome.net/cas-designer/
**Chemicals and media**		
Agarose	Sigma-Aldrich	A9539-500G
Acetic acid (glacial)	Sigma-Aldrich	1000631000
Activated charcoal	Sigma-Aldrich	C9157-500G
Bacto Dehydrated Agar	BD Biosciences	214010
Calcium Chloride ($CaCl_2$)	Sigma-Aldrich	C3881-500
DRAQ7	Thermo Fisher	D15106
Ethanol	Sigma-Aldrich	100.983
Folic acid	Sigma-Aldrich	F8758-25G
Glycerol	Sigma-Aldrich	1040572511
HEPES, ultra-pure	Biomol	5288.1
HL5 Medium including Glucose	Formedium	HLG0102
IGEPAL CA-630	Sigma-Aldrich	I8896-50M
Potassium phosphate dibasic (K_2HPO_4)	Sigma-Aldrich	P8281-500G
Potassium chloride (KCl)	Sigma-Aldrich	60130-1KG
Potassium phosphate monobasic (KH_2PO4)	Sigma-Aldrich	P0662-500G
Magnesium chloride hexahydrate ($MgCl_2 \cdot 6H2O$)	Sigma-Aldrich	M9272-1kg
Magnesium sulfate ($MgSO_4$)	Sigma-Aldrich	M2643-500G
Sodium chloride (NaCl)	Sigma-Aldrich	71380-1KG-M
Sodium phosphate monobasic (NaH_2PO_4)	Sigma-Aldrich	S3139-500G
Sodium bicarbonate ($NaHCO_3$)	Sigma-Aldrich	S5761-1KG
Penicillin G potassium salt (Benzylpenicillin potassium salt)	Sigma-Aldrich	P7794-10MU
SM Broth/5	Formedium	SMB50102
Streptomycin sulfate	Sigma-Aldrich	S9137-100G
Titriplex III (EDTA disodium salt)	Sigma-Aldrich	1084180100
Trizma base	Sigma-Aldrich	T1503-1KG
Vitamin B12 (Cyanocobalamin)	Sigma-Aldrich	V6629-250MG
Nuclease-free water (H_2O), for molecular biology (DEPC-free)	Sigma-Aldrich	W4502-10X50ML
Bacto Tryptone	Thermo Fisher	211705
Bacto Yeast Extract	Thermo Fisher	212750
**CRISPR reagents**		
Alt-R CRISPR-Cas9 tracrRNA, 20 nmol	IDT	1072533
Alt-R CRISPR-Cas9 crRNA	IDT	Custom
Alt-R S.p. Cas9 Nuclease V3, 100 µg	IDT	1081058
Alt-R Cas9 Electroporation Enhancer, 10 nmol	IDT	1075915
Alt-R HDR Enhancer V2, 30 µL	IDT	10007910
pHO4d-Cas9 (for SpyCas9 purification), see also Ref. (Hua Fu *et al*, 2014)	Addgene	67881
TrueCut Cas9 Protein v2	Thermo Fisher	A36498
**Enzymes and kits**		
QIAquick PCR Purification Kit	Qiagen	28106
Proteinase K	NEB	P8107S

Q5 Hot Start High-Fidelity 2X Master Mix	NEB	M0494L
Taq 2X Master Mix - 500 reactions	NEB	M0270L
**Plasticware/disposable material**		
Disposable loops 10µL	Thermo Fisher Scientific	10048750
Disposable loops 1µL	Thermo Fisher Scientific	10344741
Spreaders, T-shaped	VWR	612-2653
Nunc multidish 24-wells	Thermo Fisher Scientific	10604903
Nunc multidish 6-wells	Thermo Fisher Scientific	10119831
Petri dishes, steriplan, Duran 100x20	Duran	391-2840
Gene Pulser/MicroPulser electroporation cuvettes, 0.1cm gap	Bio-Rad Laboratories	1652089
Countess cell counting chamber slides	Thermo Fisher Scientific	C10312
Falcon 5 mL round bottom polystyrene test tube, without cap	Falcon	352008
Falcon round-bottom polystyrene test tubes with cell strainer snap cap, 5mL	Falcon	352235
**Equipment**		
BD FACSAria Fusion Flow Cytometer	BD Biosciences	
Gene Pulser Xcell Microbial System	Bio-Rad Laboratories	1652662
BD FACSymphony A3 Analyzer	BD Biosciences	
Axio Zoom.V16 Stereo Zoom Microscope	Zeiss	
Countess 3 starter package 1	Thermo Fisher Scientific	A49865
NanoDrop 8000 Spectrophotometer	Thermo Fisher Scientific	ND-8000-GL
BioPhotometer plus	Eppendorf AG	
C1000 Touch Thermal Cycler	Bio-Rad Laboratories	1851197

Buffers and reactions

Reagents/ solution	Final concentration	Stock reagent/ solution	Amount/ volume
10x KK2	161.6mM KH_2PO_4	KH_2PO_4 Powder	22g
	40mM K_2HPO_4	K_2HPO_4 Powder	7g
**Instructions.** Dissolve in 1L of deionized H_2O and autoclave. Store at RT.			
1x KK2-MC	1xKK2	10xKK2, autoclaved	100mL
	50µM $MgCl_2$	1 M $MgCl_2$, autoclaved	50µL
	50µM $CaCl_2$	1 M $CaCl_2$, autoclaved	50µL
**Instructions.** Mix autoclaved components, adjust volume to 1L with autoclaved deionized H_2O, filter sterilized. Store at RT.			
SM5-agar	1x SM Broth/5	SM Broth/5 powder	7.9g
	1.6% BD Bacto Dehydrated Agar	BD Bacto Dehydrated Agar powder	16g
**Instructions.** Mix reagents while stirring, adjust volume to 1L with deionized H_2O, autoclave at 110°C. Use 25mL of SM5-agar for each 10cm Petri dish. Store at 4°C. Dry plates in a laminar hood for 15 min before use.			
SM5-charcoal-agar	1x SM Broth/5	SM Broth/5 powder	7.9g
	1.6% BD Bacto Dehydrated Agar	BD Bacto Dehydrated Agar powder	16g
	0.5% Activated charcoal	Activated charcoal	5g
**Instructions.** Mix reagents while stirring, adjust volume to 1L with deionized H_2O, autoclave at 110°C. Use 1mL of SM5-agar-charcoal per well for 24-well plates and 5mL per well for 6-well plates. Store at 4°C. Dry plates in a laminar hood for 15 min before use.			
L-Broth	1% Bacto Tryptone	Bacto Tryptone powder	10g
	0.5% Bacto Yeast Extract	Bacto Yeast Extract	5g
	0.5% NaCl	NaCl Powder	5g
**Instructions.** Mix all reagents while stirring, adjust volume to 1L with deionized H_2O, and autoclave at 110°C. Store at RT.			
L-Agar	1% Bacto Tryptone	Bacto Tryptone Dehydrated	10g
	0.5% Bacto Yeast Extract	Bacto Yeast Extract Dehydrated	5g
	1% NaCl	NaCl Powder	10g
	1.5% BD Bacto Dehydrated Agar	BD Bacto Dehydrated Agar, powder	15g
**Instructions.** Mix all reagents while stirring, adjust volume to 1L with deionized H_2O, and autoclave at 110°C. Use 25mL L-Agar for each 10cm petri dish. Store at 4°C. Dry plates in a laminar hood for 15 min before use.			
H50	50mM KCl	1M KCl, autoclaved	50mL
	20mM HEPES pH 7.0	400mM HEPES pH7, filter sterilized	50mL
	10mM NaCl	1M NaCl, autoclaved	10mL
	5mM $NaHCO_3$	500mM $NaHCO_3$, filter sterilized	10mL
	1mM NaH_2PO_4	100mM NaH_2PO_4, autoclaved	10mL
	1 mM $MgSO_4$	1M $MgSO_4$, autoclaved	1mL
**Instructions.** Mix components, adjust volume to 1L with autoclaved deionized H_2O and filter sterilize. Store at 4°C.			
Quick Genomic DNA extraction buffer	0.5x Taq buffer	10x Taq buffer	1µL
	0.5% IGEPAL CA-630	IGEPAL CA-630 liquid	0.1µL
	50ng/µL Proteinase K	20mg/mL Proteinase K, solution	0.05µL
	H_2O	Nuclease-Free H_2O	18.85µL

**Instructions.** Mix all components on ice, and use 20µL per extraction. Prepare freshly before use.			
Quick Genomic DNA extract	Quick Genomic DNA extraction buffer	Quick Genomic DNA extraction buffer	20µL
	3-5 sori of monoclonal species	Sori of fruiting bodies growing in SM5-agar plates	3-5 sori
**Instructions.** Prepare Quick genomic DNA extraction buffer freshly. Make 20µL aliquots in PCR tubes. With a 10µL tip, pick 3-5 sori and dip them in the extraction buffer. Incubate at 56°C for 45 min and 95°C for 10 min. Store at 4°C for up to 3 days.			
Clone confirmation PCR reaction	1x Taq polymerase	Taq 2X Master Mix	10µL
	0,2µM Forward primer	10µM Forward primers, in 10mM Tris-Cl, pH 8.5	0.4µL
	0.2µM Reverse primer	10µM Reverse primers, in 10mM Tris-Cl, pH 8.5	0.4µL
	H_2O	Nuclease-free H_2O	To 20µL
	Quick Genomic DNA extract	Quick Genomic DNA extract	2µL
**Instructions.** Set up PCR reaction on ice by mixing all components. A master mix can be prepared for several samples, and 18µL aliquots prepared in PCR tubes. Two microliter of Quick Genomic DNA extract are added to each reaction. PCR cycle: 1x initial denaturalization cycle at 95°C for 2 min, 35x amplification cycles at 95°C for 30 sec, T_a depending on primers for 20 sec, 68°C for 1 min, and 1x final extension cycle at 68°C for 10 min. Run 2µL PCR product, with 2µL 6x loading dye and 6µL nuclease-free H_2O, in a 1% agarose gel. Purify the remaining 18µL with a Qiagen QIAquick PCR purification column, elute with 25µL EB and send for Sanger sequencing.			
1000x Pen/Strep for HL5	100mg/mL Streptomycin sulfate	Streptomycin sulfate, powder	1g
	70mg/mL Penicillin G potassium salt	Penicillin G potassium salt (Benzylpenicillin potassium salt), powder	0.7g
**Instructions.** Mix all components and adjust to 10mL with autoclaved deionized H_2O. Filter sterilize with 0.22µm syringe filters. Make 1mL aliquots and store at -20°C. Add to HL5 just before use to a final concentration of 1x.			
10000x VitB12 / Folic acid	2mg/mL Folic acid	Folic acid, powder	20mg
	6mg/mL Vitamin B12	Vitamin B12 (Cyanocobalamin), powder	60mg
**Instructions.** Mix all components and adjust to 10mL with autoclaved deionized H_2O. Adjust pH to 7.0 with NaOH. Filter sterilize with 0.22µm syringe filters. Make 0.1mL aliquots and store at -20°C. Add to HL5 just before use to a final concentration of 1x.			
HL5 broth	35.5g/L HL5	HL5 Medium including Glucose, powder	35.5g
**Instructions.** Mix with deionized H_2O while stirring, adjust volume to 1L, and autoclave at 110°C. Store at 4°C.			
HL5+FAB	HL5 broth	HL5 broth, autoclaved	Required volume for the experiment
	1x Pen/Strep for HL5	1000x Pen/Strep for HL5, solution	1:1000 (v:v)
	1x VitB12/Folic acid	10000x VitB12/Folic acid, solution	1:10000 (v:v)
**Instructions.** Warm the needed volume of 1x HL5 Broth to 22°C and add Pen/Strep and VitB12/Folic acid to a final concentration of 1x just before use.			
RNP complex	12µM Cas9	~15µg/µL Cas9 in 20mM HEPES pH 7.5, 500mM KCl, 10% glycerol. Produced by EMBL PeP-core facility[9]	1.31µL[10]

[9] The best results are obtained with ultrapure WT SpyCas9 (Appendix Figure S2). However, good efficiencies are also obtained using a quick 1-day purification protocol. TrueCut Cas9 Protein v2 (Thermo Fisher, A36498) is the best-performing commercially available SpyCas9 we tested (Appendix Figure S2).
[10] Volume might change depending on batch (depending on SpyCas9 activity).

	15µM gRNA complex	50 µM gRNA complex (100µM Alt-R CRISPR-Cas9 tracrRNA and 100µM Alt-R CRISPR-Cas9 crRNA in nuclease-free H_2O, pre-annealed)	3µL[11]
	H_2O	Nuclease-free water	Up to 10µL

Instructions. For each transfection, mix 1.5µL of 100µM Alt-R CRISPR-Cas9 tracrRNA and 1.5µL of 100µM Alt-R CRISPR-Cas9 crRNA in a PCR tube, and anneal by heating at 95ºC for 5 min, and slowly cooling down (-1ºC/cycle, 20sec/cycle). Scale up depending on the number of transfections with the same crRNA. The 50µM gRNA complex can be stored at -20ºC for up to 1 year. Mix components in a 1.5mL tube at RT following the indicated order, by slowly adding each component while swirling the tip, and pipetting once up and down after the addition of each component.

Transfection mix	RNP complex (see above)	12µM Cas9 / 22µM Alt-R CRISPR-Cas9 tracrRNA / 22µM Alt-R CRISPR-Cas9 crRNA	10µL
	Donor oligo, as indicated	100µM oligo, in nuclease-free H_2O	As indicated
	Donor PCR product, as indicated	1µg/µL donor PCR product	As indicated
	EE	100µM Alt-R Cas9 Electroporation Enhancer (EE), in nuclease-free H_2O	Optional[12]
	H50	H50 buffer at RT	Up to 100µL in small volume increments
	1.25-5·10^6 Dictyostelid cells	Cells washed in ice-cold H50	Cell pellet

Instructions. To 10µL of RNP complex, add donor oligos, donor PCR products and/or EE when indicated. Bring up volume to 100µL by adding H50 buffer in small increments (for example 25+25+40µL). Spin down aliquots of cells, remove supernatant, and resuspend pellet with 100µL RNP complex in H50 buffer. Transfer 80µL to a pre-chilled electroporation cuvette. After electroporation, transfer cells to growth media as indicated.

Donor PCR product	1x Q5 Hot Start High-Fidelity	Q5 Hot Start High-Fidelity 2X Master Mix	200µL
	0.5µM Forward Primer	10µM Forward Primer in 10mM Tris-Cl, pH 8.5	20µL
	0.5µM Reverse Primer	10µM Reverse Primer in 10mM Tris-Cl, pH 8.5	20µL
	0.2ng/µL plasmid DNA Template	5ng/µL plasmid DNA Template in 10mM Tris-Cl, pH 8.5	16µL
	H_2O	Nuclease-Free H_2O	Up to 400µL

Instructions. For a transfection with 10µg of donor PCR product, prepare 400µL of donor PCR reaction by mixing all the components on ice. Split in 8x 50µL in PCR tubes, and use the following PCR cycle: 1x Initial Denaturalization cycle at 98ºC for 30s, 30x Amplification cycles at 98ºC for 10 sec, 61.5ºC for 10 sec, 72ºC for 30 sec, and 1x Final Extension cycle at 72ºC for 4 min. Pool together 400µL of PCR reaction and purify using one Qiagen MiniElute column. Elute with 12µL nuclease-free H_2O. Quantify using a NanoDrop 8000 Spectrophotometer. T_a might have to be optimized for different templates/primers.

DRAQ7	3µM DRAQ7	DRAQ7 Dye (0.3mM in aqueous buffer)	1:100 (v:v)

Instructions. Add 1:100 (v:v) to cells in KK2-MC buffer and briefly vortex. Incubate 5 min before analyzing cells with flow cytometer.

50xTAE	0.5M Trizma base	Trizma base, powder	242g
	0.05M EDTA, pH 8.0	0.5M EDTA, pH 8.0, autoclaved	100mL
	57% Acetic acid	Acetic acid (glacial), 100% anhydrous	57mL

Instructions. Mix all components and adjust to 1L with deionized H_2O.

1xTAE	1xTAE	50xTAE	20mL

[11] Optional: pre-annealing of tracrRNA and crRNA to generate a gRNA complex can be omitted, resulting in slightly lower efficiencies (Appendix Figure S2).
[12] Comes with the risk of inserting EE derived DNA sequences into your target site.

**Instructions.** Add 20mL 50xTAE to 980mL deionized H_2O. Store at RT.			
1% agarose gel	1% agarose	Agarose, powder	1g
	1xTAE	1xTAE	100mL
**Instructions.** Add agarose to 100mL 1xTAE while stirring. Microwave for 1 min, stir, microwave for 1 min. Let it cool down for 5 min. Poor on a tray, add 3μL SYBR Safe, gently mix evenly, place a comb, and let the gel polymerase for 20 min. After loading, run the gel for 30-40 min at 100-120V.			

Part 1. CRISPR-Cas9 editing protocol with RNP complex in *D. discoideum*.

Step 1. Identifying good CRISPR-Cas9 targets.

The RNP complex consists of the endonuclease Cas9, crRNA and tracrRNA. The 20nt spacer sequence of crRNA complements the genomic target site. The target site also needs an adjacent PAM sequence to be recognized by Cas9. We use Streptococcus pyogenes Cas9 (SpyCas9) that recognizes the PAM sequence 'NGG'. Before we can start gene editing, we should first identify good CRISPR-Cas9 target sites in the genome, which allows us to design and order a custom crRNA. The other components of the RNP complex are invariable (SpyCas9 and tracrRNA).

Tools to identify CRISPR-Cas9 targets.

- *There are several tools available for identifying potential CRISPR targets. By providing both the species of interest and gene of interest (GOI), these tools will identify unique CRISPR targets within the GOI that have no or minimal off-target hits elsewhere in the genome. Popular tools are for example Cas-designer (http://www.rgenome.net/cas-designer/)* (Bae *et al*, 2014a, 2014b; Park *et al*, 2015) *and CRISPOR (http://crispor.tefor.net/)* (Concordet & Haeussler, 2018)*. The reference genome of D. discoideum is available for both these tools.*

Tools to simulate CRISPR-Cas9 edit.

- *Before starting gene editing, we recommend simulating the CRISPR-Cas9 edit with a cloning software, such as SnapGene or Benchling, to ensure the correct crRNA and donor template design for CRISPR-Cas9 editing. In this protocol, we use SnapGene for that purpose (https://www.snapgene.com/).*

For introducing knock-out or knock-in mutations, it is important to use the genomic sequence associated with the GOI for designing the crRNA, while making sure that the insertion site is present in an exonic region (i.e., present in the coding DNA sequence). In addition, the cutting site should be as close as possible to the desired insertion site. We recommend a window of +/-10bp from the cutting site (Paix *et al*, 2019)*. In the current study, we always cut within +/-3bp from the desired insertion site.*

1.1. Identifying a CRISPR-Cas9 target for a GOI.
- Find both the genomic sequence and the coding DNA sequence (CDS) associated with a GOI on dictyBase (http://dictybase.org/).
- Open Cas designer and fill out the below information.
    - PAM type: SpyCas9 from *Streptococcus pyogenes*: 5'-NGG-3'
    - Target genome: Others, *Dictyostelium discoideum* (dictyBase)
    - Target sequence: Genome sequence containing the CDS[13] of a GOI.
    - crRNA length: 20.
- Identify spacer sequences that are associated with best target sites, ideally:
    - No off-targets hits for spacer sequences with 0, 1 or 2 mismatches.
    - Out-of-frame score of ≥66.
    - GC content >20% (without the PAM sequence).
    - No AT rich or repetitive sequences[14].

---

[13] Cas-designer only allows up to 1000bp sequences. Longer genes can be split into smaller fragments. Targeting the 5' end of the CDS works best for creating complete knock-outs. Targeting the 3' end might result in only partial deletion of the protein.

[14] Cas designer tool returns 30 possible hits per search. Since *Dictyostelid* genomes are AT rich and repetitive, when there are several spacer sequences possible, choosing a spacer associated with a less repetitive sequence can reduce off-target effects and simplify downstream PCRs to confirm gene edits.

- Choose two or three spacer sequences targeting a GOI[15]. Below we show three spacer sequences we identified for *mCherry* in *D. discoideum* AX2 *act5::mCherry* (visualized in SnapGene).

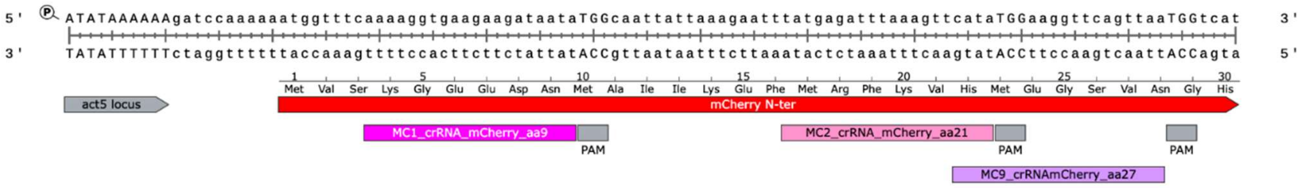

1.2. Order and prepare crRNA.

- When ordering from IDT, enter the 20nt spacer sequence upstream from the PAM. The ordering tool will automatically convert the DNA sequence into an RNA sequence (see also Figure 1A). In the case of MC1 targeting *mCherry*, the spacer sequence for the crRNA is:

```
Target sequence   5′-...GGTTTCAAAAGGTGAAGAAGATAATATGGCAATTA...-3′
Ordered crRNA               5′-aaaaggtgaagaagataata-3′
```

- 2nmol crRNA is sufficient for 8 or 9 experiments.
- Resuspend crRNA in nuclease-free H_2O to a final concentration of 100µM.

1.3. Order and prepare tracrRNA.

- As the same tracrRNA can be used for all CRISPR-Cas9 experiments, we recommend ordering a larger quantity: 20nmol of tracrRNA is sufficient for 80-90 experiments.
- Resuspend tracrRNA in nuclease-free H_2O to a final concentration of 100µM.

Step 2. Designing donor oligos and donor PCR products.

To introduce precise genome edits using HDR, a donor oligo or donor PCR product that serves as a repair template is needed. Similar to designing crRNAs, when designing a repair template, it is important to use the genomic sequence of the target site, and not the CDS, since target sites can be close to exon/intron junctions. Genome editing is also possible without repair template, but will result in less-efficient NHEJ-mediated indels. Knock-out of a GOI can be efficiently produced with donor oligos with 1 or 4bp insertions that result in a frameshift. Short sequences, such as FLAG-tags, HA-tags or Myc-tags can also be introduced using single-stranded donor oligos. For knock-outs, we find that donor oligos with a 37bp insertion (coding for HA-tag, a stop codon and a frameshift mutation) and 28bp homology arms give the best efficiencies (Figure 1). To introduce larger inserts, to for example generate knock-ins, PCR products can be used as repair templates for HDR. The donor PCR product consists of the insertion sequence (for example the CDS of a fluorescent protein) and ~30bp homology arms (see Figure 2 and Paix et al. 2017). The best efficiencies are obtained for lower insertion sizes, ideally smaller than 1000bp. Similarly to HDR with donor oligos, for producing knock-ins by HDR with a donor PCR product, the insertion site should be as close as possible to the cutting site. It is also important that the CRISPR-Cas9 target site is removed after creating the knock-in to ensure single edits only. The target site can be removed by either removing the PAM sequence or removing complementarity to the crRNA spacer sequence.

[15] Make sure the cutting sites of the selected spacers are on the CDS of a GOI (and not in an intronic region).

2.1. Design of donor oligos with 1bp insertion for producing knock-outs.

- Design the donor oligo with a 1bp insertion to introduce a frameshift mutation in the GOI with 28bp homology arms flanking the insertion site[16]. If possible, search for a 1bp insertion near the cutting site that will introduce a stop codon[17]. Below is the donor oligo we used for introducing a 1bp insertion in *mCherry* 1bp away from the cutting site, causing both a stop codon and frameshift mutation.

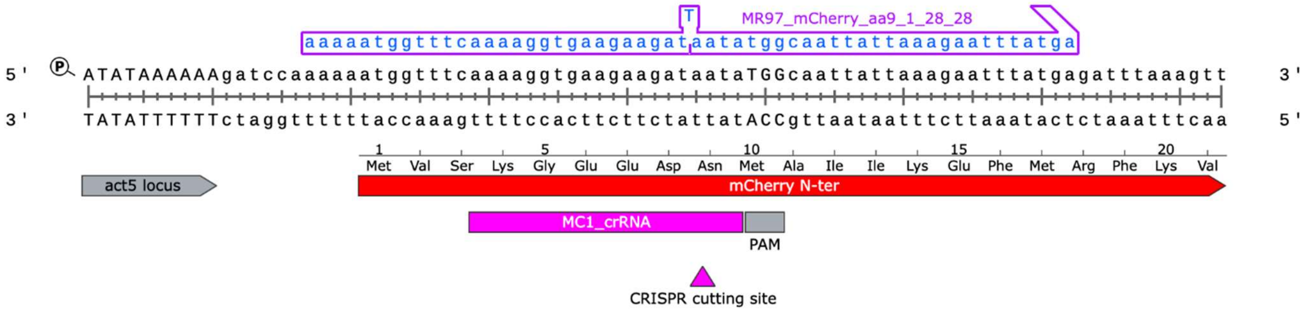

- Simulate the CRISPR-Cas9 edit using your crRNA and designed donor oligo in a cloning software (e.g., SnapGene). Below is the final product of the CRISPR-Cas9 edit with a 1bp insertion for our *mCherry* target. By inserting 1bp we introduce several stop codons in the amino acid sequence of *mCherry*.

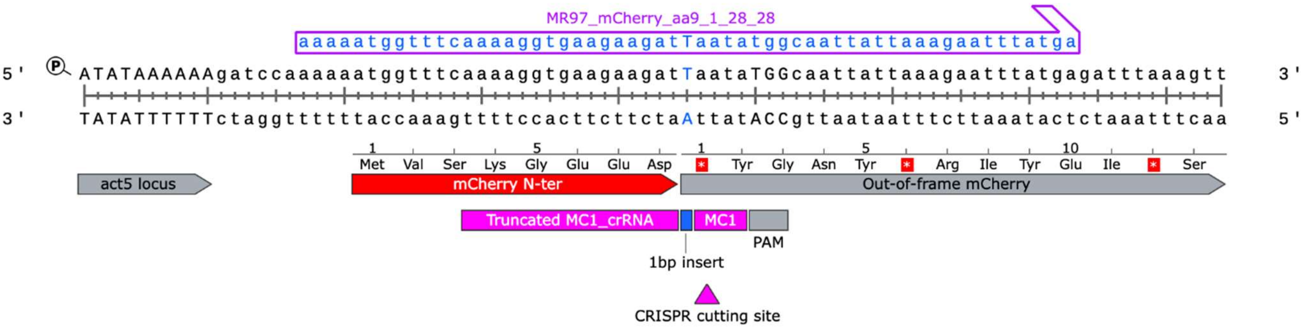

2.2. Design of donor oligos for introducing tags.

- Design a donor oligo with the sequence of the tag of interest and 28bp homology arms flanking the insertion site.
- To knock-out the target gene, add a stop codon and an additional 1bp to produce a frameshift mutation. In the example below we introduce an HA-tag, a stop codon and a frameshift mutation to knock-out *mCherry*.

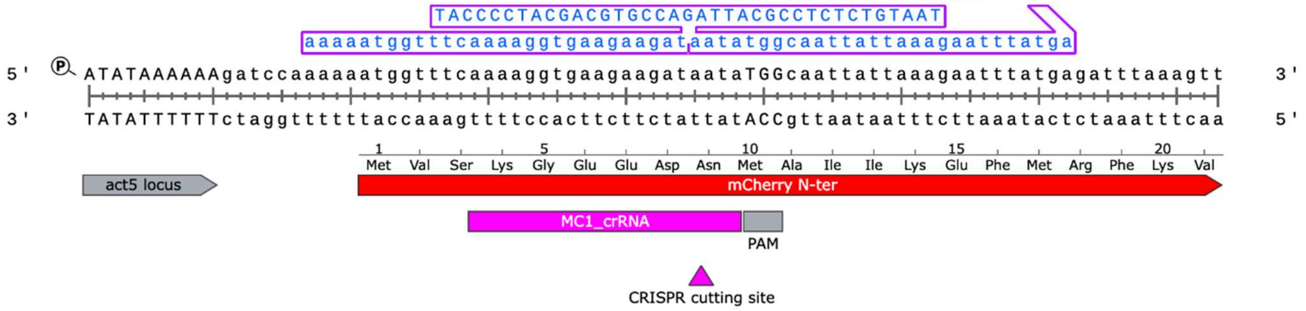

[16] SpyCas9 cuts the genome between the 3rd and 4th bp upstream of the PAM. We recommend designing an insertion that falls within +/-10bp from the cutting site (Paix *et al*, 2019). In the example above, the insertion site is 1bp from the predicted cutting site.

[17] Frameshift mutations will lead to several random stop codons downstream of the insertion thereby ensuring proper knock-out.

- Simulate the CRISPR-Cas9 edit in SnapGene to ensure that the tag sequence is in frame, and that stop codons have been introduced when knocking-out the target gene.

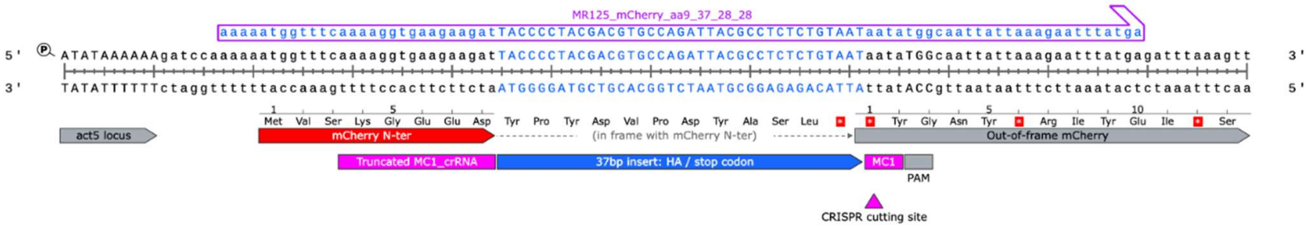

2.3. Design of donor PCR products for generating knock-in mutants.

- Design forward and reverse primers with 18-22 nucleotides of homology to the template DNA (e.g., pMG005) and 30-40bp overhangs with homology to the genomic sequences flanking the insertion site (i.e., homology arms). We recommend having a G or C at the 3'end of each primer. The T_m of the primer pair should be similar (<3°C difference). We usually target for a T_m of 59°C and adjust the length of the homology arms accordingly. In the example below, we create an in-frame knock-in mutation in *mCherry* using *mNeonGreen-P2A* as template (pMG005). The final product expresses both *mNeonGreen* and *mCherry* from the same promoter (Zhu *et al*, 2023). In our example, the forward/reverse primers were designed with 34/37bp homology arms (blue sequence) and 19/20bp annealing (black sequence) to the template (pMG005), respectively.

```
AP1169:  5'GATCCAAAAAATGGTTTCAAAAGGTGAAGAAGATGTATCTAAGGGAGAAGAAG 3'
AP1170:  5'CTTTAAATCTCATAAATTCTTTAATAATTGCCATATTAGAACTAGATCCACTAGGTC  3'
```

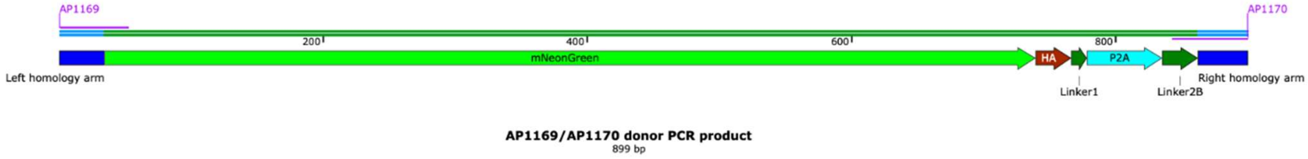

- Using a cloning software, simulate the PCR product and CRISPR-Cas9 edit to ensure the crRNA and donor PCR product are designed properly and no unintentional frameshift mutations or stop codons are introduced. For our example, the knock-in is expected to look as follows:

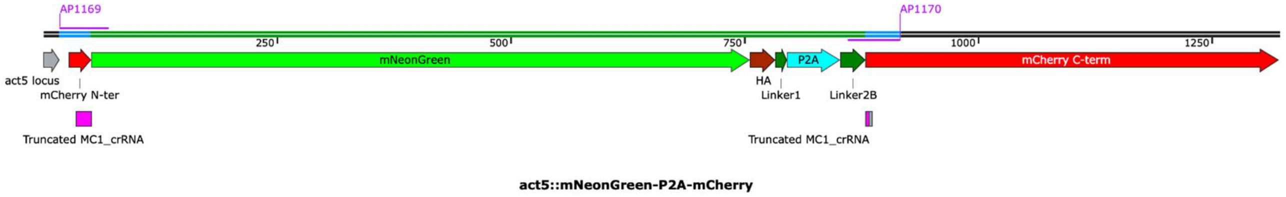

2.4. PCR

- Prepare ~10µg of PCR product[18] for each transfection, in 8x50µL PCR reactions and run the PCR using the following conditions:

[18] 10µg of donor DNA product gives good knock-in efficiencies (Figure 2), although lower amounts are effective as well (Figure 3).

Template	Primer fwd	Primer rev	PCR product size (bp)
pMG005	AP1169	AP1170	899

Reagent	Volumes for 8x50µL
Q5 Hot Start High-Fidelity 2X Master Mix	200µL
10µM forward primer	20µL
10µM reverse primer	20µL
5ng/µL template DNA	16µL
Nuclease-free H_2O	Up to 400µL (Split in 8 reactions)

Step	Temperature	Time
Initial Denaturation	98°C	30 sec
Amplification: 30 cycles	98°C	10 sec
	61.5°C[19]	10 sec
	72°C	30 sec
Final Extension	72°C	4 min
Hold	4–10°C	

- Run 2µL of PCR product from one of the tubes (with 2µL 6x loading dye and 6µL H_2O) in a 1% agarose gel to confirm the PCR was successful.
- In the meantime, pool together all PCR products (8x50µL) and purify them using a single Qiagen QIAquick PCR purification column. In brief:
    - Pool together 400µL PCR reaction in a 15mL tube.
    - Add 2000µL PB buffer to the tube.
    - Add 20µL 3M NaOAc to the tube.
    - Add 750µL PCR product/PB buffer to the column.
    - Spin at 13000xg for 30 sec.
    - Discard flowthrough.
    - Repeat using the same column, until all the PCR product has been applied to the same column.
    - Add 750µL PE buffer.
    - Spin at 13000xg for 30 sec.
    - Discard flowthrough.
    - Spin at 13000xg for 2 min to eliminate all the ethanol.
    - Transfer the column to a clean 1.5mL tube.
    - Let the column stand for 2 min.
    - Add 22µL of nuclease-free H_2O to the center of the column.
    - Let the column stand for 2 min.
    - Spin at 13000xg for 1 min.
    - Discard column and recover DNA.
    - Use 1µL of purified DNA to measure the concentration by NanoDrop.
    - We expect to have a concentration of at least 1µg/µL.
    - The PCR product can be stored at -20ºC until further use.

[19] T_a might have to be adjusted depending on the primers and the amplified sequence. We design all our primers to have a T_m of ~59ºC, and successfully use a T_a of 61.5ºC during PCR amplification.

Step 3. Culturing axenic and non-axenic strains of *D. discoideum.*

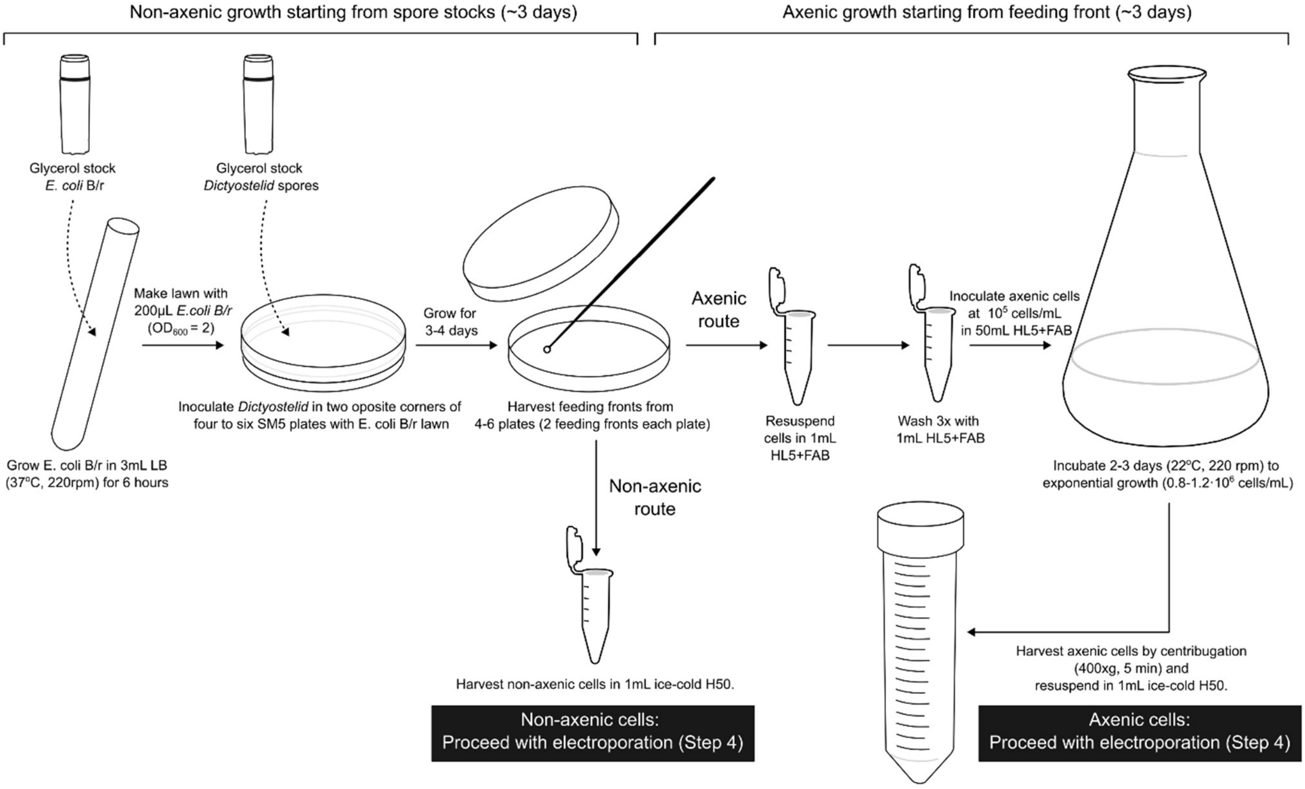

3.1. Non-axenic growth of *D. discoideum* on *E. coli* B/r.

- Prepare *E. coli* B/r.
    - Inoculate 50mL of LB with *E. coli* B/r from glycerol stock.
    - Shake O/N at 37°C 220 rpm[20].
    - Transfer culture to a 50mL falcon tube.
    - Pellet cells (4000xg, 5 min) and remove supernatant.
    - Wash pellet twice with 1mL KK2 buffer.
    - Resuspend pellet in 1mL KK2.
    - Measure culture density (OD_{600}).
    - Concentrated *E. coli* B/r in KK2 can be stored at 4°C for up to one week.
- Prepare four to six SM5-agar plates for each strain.
    - Dry plates in the hood with the lid off for 15 min.
    - Adjust density of *E. coli* B/r in KK2 from previous step to OD_{600}=2 with KK2 buffer.
    - Spread 200µL of *E. coli* B/r at OD_{600}=2 on the plates.
    - Distribute evenly with a spreader to form a bacteria lawn as food source.
    - Let it dry in the hood for 3-5 min.
- Inoculate *D. discoideum* strain directly from a glycerol stock[21].
    - Take glycerol stock from -80°C on dry ice.
    - With a loop or pipette tip, take some spores from the glycerol stock.
    - Inoculate two opposite corners of the plate.
- Incubate at 22°C for:
    - 2-3 days for harvesting both feeding fronts from the same plate.
    - 5-7 days for harvesting spores from a lawn of fruiting bodies.

---

[20] Alternatively, a 3mL culture can be grown at 37°C (220 rpm) for 6 hours on the same day.

[21] Our glycerol stocks are made from fruiting body spores.

3.2. Axenic growth of *D. discoideum* in HL5.

- Harvest *D. discoideum* cells from SM5-agar plates.
    - With a 10µL loop, harvest cells from the feeding fronts from 4 to 6 SM5-agar plates (2 feeding fronts from each plate).
    - Resuspend cells in 1mL of HL5+FAB in a 1.5mL tube.
    - Spin down cells (400xg, 2 min) and remove supernatant.
    - Wash cells twice with 1mL HL5+FAB.
    - Resuspend cells in 1mL HL5+FAB.
    - Dilute cells 1/10 in HL5+FAB.
    - Measure cell density using a hemocytometer or an automated cells counter (e.g., Countess III cell counter).
- Inoculate an HL5+FAB culture.
    - Prepare 50mL of HL5+FAB in a 250mL flask (sufficient for 5-10 transfections).
    - Add cells to have a final density of 10^5 cells/mL.
    - Grow cells at 22°C (180rpm) for 2-3 days until a density of $0.8\text{-}1.2 \cdot 10^6$ cells/mL (exponential growth).

Step 4. Transfecting cells with RNP complex by electroporation.

4.1. Prepare non-axenic cells for electroporation.

- Prepare 4 to 6 SM5-agar plates with the cells of interest as indicated in Step 3.1.
- Incubate plates for 2-3 days or until there are two visible feeding fronts per plate.
- Harvest cells from the feeding fronts of 4 to 6 plates with a 10µL inoculation loop.
- Transfer them to a 1.5mL tube with 1mL of ice-cold H50 buffer.
- Spin cells down (400xg, 2 min) and remove supernatant.
- Wash cells with 1mL of ice-cold H50 twice.
- Resuspend cells in 1mL of ice-cold H50.
- Make a 1/100 dilution and count cells.
- Make aliquots of $1.25\text{-}5 \cdot 10^6$ cells for each transfection[22]. The volume is not important, because cells will be pelleted again later. Keep on ice.
- Proceed with Step 4.3.

4.2. Prepare axenic cells for electroporation.

- Count cells from Step 3.2 daily until they reach exponential phase.
    - Transfer 1mL of culture with serological pipette to a 1.5mL tube.
    - Count cells as in previous step. If they are within the right interval of $0.8\text{-}1.2 \cdot 10^6$ cells/mL, proceed with the next step. If cells are overgrown (over $2 \cdot 10^6$ cells/mL), back dilute them to $5 \cdot 10^5$ cells/mL, and let them grow for another day. They should be ready on the following day.
- Prepare aliquots for transfection.
    - Transfer 45mL of cell suspension ($0.8\text{-}1.2 \cdot 10^6$ cells/mL) to a 50mL tube.
    - Spin cells down (400xg, 5 min) and remove supernatant. Be careful not to remove the pellet as it does not firmly attach to the bottom of the tube.
    - Add 1mL of ice-cold H50 and transfer cells to a 1.5mL tube.
    - Spin cells down (400xg, 2 min) and remove supernatant
    - Wash cells with 1mL of ice-cold H50 twice.
    - Resuspend cells in 1mL of ice-cold H50.
    - Make a 1/100 dilution and count cells.

[22] We typically use $2.5 \cdot 10^6$ cells for each transfection. See Appendix Figure S1A.

- Make aliquots of 1.25-5·10^6 cells for each transfection. The volume is not important, because cells will be pelleted again later. Keep on ice.
- Proceed with Step 4.3.

4.3. Prepare plates for growth after transfection.

- For analysis:
  - For non-axenic cells[23]:
    - Option 1: prepare 24-well plates with 1mL *E. coli* B/r suspension (OD_{600}=1-2 in KK2-MC) per well for each transfection.
    - Option 2: for each transfection, prepare one 10 cm petri dish with 20mL SM5-agar with an *E. coli* B/r lawn.
  - For axenic cells, prepare 24-well plates with 1mL HL5+FAB per well for each transfection.
- For sorting:
  - For non-axenic cells:
    - Option 1: prepare 6-well plates with 5mL *E. coli* B/r suspension (OD_{600}=1-2 in KK2-MC) per well for each transfection.
    - Option 2: for each transfection, prepare one 10 cm petri dish with 20mL SM5-agar with an *E. coli* B/r lawn.
  - For axenic cells, prepare 6-well plates with 5mL HL5+FAB per well for each transfection.
- Keep on the side.

4.4. Prepare the RNP complex.

- Anneal tracrRNA and crRNA[24] by mixing the following components in a 0.2mL PCR tube to prepare the gRNA complex at a final concentration of 50µM. Scale up depending on the number of reactions.

Reagent / solution	Amount/transfection
100µM tracrRNA	1.5µL
100µM crRNA	1.5µL

- Heat at 95°C for 5 min.
- Cool down slowly to RT (-1°C/cycle, 20sec/cycle).
- The gRNA complex can be stored at -20°C for up to 1 year.
- Assemble the RNP complex by mixing the following reagents at RT in the order specified by the table in a 1.5mL tube. Add the reagents very slowly, swirling the tip into the mix, and gently pipetting up and down once or twice after the addition of each reagent.

[23] Recovering cells in liquid media (option 1) allows to easily and quickly harvest cells before flow cytometry analysis and/or sorting. However, recovery in SM5-agar media (option 2) improves gene editing efficiencies (see Figure 3).

[24] Pre-annealing of the crRNA and tracRNA results in better efficiencies (Appendix Figure S2), and therefore we recommend including this step. However, this step can be eliminated, and the crRNA and tracrRNA can be directly added to the Cas9 protein.

Reagent / solution	Amount/transfection	Final concentration
~15μg/μL (~90μM) SpyCas9[25]	1.31μL	~12μM
50μM gRNA complex	3μL	15μM
Nuclease-free H_2O	Up to 10μL	

4.5. Transfection mix in electroporation buffer.

- Add the following reagents to the RNP mix as indicated.

Reagent / solution	Amount/transfection with donor oligo	Amount/transfection with donor PCR product	Final concentration
RNP complex	10μL	10μL	
100μM donor oligo	0-4.8μL	NA	0-4.8μM
1μg/μL donor PCR product	NA	0-20μL	0-20μg/100μL[26]
H50 buffer	Up to 100μL	Up to 100μL	

- Add H50 buffer to the RNP complex in small volume increments (for example, 15+25+40μL) to a final volume of 100μL.
- Combine cells and transfection mix[27].
    - Spin down aliquot of cells ($1.25\text{-}5\cdot10^6$ cells; 400xg, 2 min) and remove supernatant.
    - Resuspend cell pellet by adding 100μL of RNP complex and gently pipetting up and down once or twice.
    - Keep cell suspension with RNP complex on ice.

4.6. Electroporation.

- Pre-chill electroporation cuvettes (1mm gap, Bio-Rad).
- Transfer 80μL of each sample to ice-cold electroporation cuvette.
- Dry the sides of the cuvette with a kimwipe.
- Electroporate using a Gene Pulser Xcell Microbial System.
- Electroporation settings:
    - Exponential protocol
    - Voltage (V): 750
    - Capacitance (μF): 25
    - Resistance (Ohms): Infinite
    - Cuvette (mm) :1
    - Number of pulses: 1
- Place cuvette on ice immediately after electroporation.

[25] The best results are obtained with ultrapure WT SpyCas9 (Appendix Figure S2). However, good efficiencies are also obtained with a regular 1-day purification protocol (see Methods for both quick and extended purification protocols). TrueCut Cas9 Protein v2 (Thermo Fisher, A36498) is the best-performing commercially available SpyCas9 we tested (see Appendix Figure S2).

[26] In our example, where we knock-in *mNeonGreen-P2A* into *mCherry*, the donor PCR fragment is 899bp long. Therefore, 1μg of PCR product = 1.8pmols, and 1μg/100μL = 1.8μM.

[27] Note that for combinatorial knock-ins or knock-outs, independent transfection mixes can be prepared, and then mixed at a 1:1 ratio before adding them to the cells and proceeding with Step 4.6.

4.7. Grow cells after electroporation.

- Let the electroporated cells stand on ice for 5 min.
- For analysis, transfer cells to plates prepared in Step 4.3:
  - For non-axenic cells[28]:
    - Option 1: transfer 10-20µL to 24 well plate with 1mL *E. coli* B/r suspension (OD_{600}=1-2 in KK2-MC) per well.
    - Option 2: spot 10µL of electroporated cells on the center of an SM5-agar plate with an *E. coli* B/r lawn.
  - For axenic cells, transfer 10-20µL to 24 well plate with 1mL HL5+FAB per well.
- For sorting, transfer cells to plates prepared in Step 4.3:
  - For non-axenic cells:
    - Option 1: transfer 60-80µL to a well of a 6-well plate prepared in Step 4.3 with 5mL *E. coli* B/r suspension (OD_{600}=1-2 in KK2-MC) per well.
    - Option 2: make 2 spots of 20 µL on two opposite sides of an SM5-agar plate with an *E. coli* B/r lawn.
  - For axenic cells, transfer 60-80µL to a well of a 6-well plate with 5mL HL5+FAB per well.
- Incubate cells for 1-3 days.
- For transfection recovering in liquid media, replace media every day.
- Proceed to analysis by flow cytometry or clone isolation by FACS sorting.

Step 5. Flow cytometry analysis.

When generating a fluorescent knock-in mutant at the act5 locus, fluorescent cells can be detected a few hours after transfection. Flow cytometry can therefore be done within a day. When knocking out a fluorescent protein, like the mCherry gene in our example, loss of fluorescence intensity in cells is also visible within one day, but it becomes more apparent as the fluorescent protein is diluted out and degraded. We therefore typically analyze knock-out and knock-in efficiencies 2 days post-transfection.

5.1. Prepare cells for flow cytometry analysis.

- Harvest transfected cells 1-3 days after transfection from 24-well plate.
  - For cells growing in liquid media (HL5 or *E. coli* B/r suspension, OD_{600}=1-2 in KK2-MC):
    - Remove media with a cell culture vacuum aspirator (or a pipette).
    - Add 400µL KK2-MC buffer.
    - Harvest cells by gently pipetting them up and down.
    - Transfer 200µL of cells to a 5mL Falcon round-bottom tube.
    - Add 2µL DRAQ7.
    - Briefly vortex.
    - Incubate 5-10 min.
  - For cells growing on SM5-agar plates:
    - Using a 10µL loop, harvest the feeding front (vegetative cells) and transfer it to 1mL KK2-MC buffer.
    - Spin down at 400xg, 2 min.
    - Resuspend in 400µL KK2-MC buffer.
    - Transfer 200µL of cells to a 5mL Falcon round-bottom tube.

[28] Option 1 allows to easily and quickly harvest cells before flow cytometry analysis and sorting. However, option 2 improves gene editing efficiencies.

- Add 2µL DRAQ7.
- Briefly vortex.
- Incubate 5-10 min.

5.2. Analysis by flow cytometry.
- Analyze cells in BD FACSymphony A3 analyzer with BD FACSDiva software Version 9.1.2 (or another available flow cytometer).
  - For the FACSymphony, we use the following settings.
    - FSC: 100V
    - SSC: 150V
    - 488-530_30: 285V
    - 561-610_20: 434V
    - 640-670_30: 500V
    - Flow rate lower than 500 cells/sec.
  - When possible, include both negative and positive controls for gating purposes.
- Visualize results with FlowJo or other available tools.
  - Gate cells using FSC and SSC channels. Particularly important for gating against bacterial cells when using non-axenic cultures.
  - Gate for living cells using 640-670_30. Living cells are impermeable to DRAQ7, and are therefore negative in the 640-670_30 channel.
  - Gate for singlets using FSC-A vs FSC-H.
  - Gate fluorescent and non-fluorescent cells based on 561-610_20 (mCherry-/mCherry+ cells) or 488-530_30 (mNeonGreen-/mNeonGreen+ cells) channels. Use controls to confirm appropriate gating.

Step 6. Isolation of gene-edited cells using cell sorting.

When knocking-in a fluorescent protein under the control of a promoter that is expressed during growth (i.e., vegetative life stage), like the act5 gene, expression can be detected within a few hours after transfection. We therefore normally sort cells one or two days post-transfection. When knock-in mutants have a growth defect, sorting cells earlier might increase efficiency.

6.1. Cell sorting.
- Prepare 24-well plates.
  - Add 1mL autoclaved SM5-charcoal-agar[29] to each well using a multi-dispenser pipette[30] and let it solidify with the lid off for 20-30 min under a vertical flow hood. Plates can be stored at 4°C for up to 1 month.
  - On the day of sorting, add 25µL of *E. coli* B/r cell suspension ($OD_{600=}$0.33 in KK2-MC buffer) to each well. Spread the cells using a heat-bended glass Pasteur pipette.
  - Let it dry for 10-25 min.

[29] We add charcoal to the SM5-agar media to have black growth media and reduce autofluorescence of the media during imaging of the plates to identify positive clones by fluorescence microscopy (see below).
[30] Adding 1mL of agar-media with a multi-dispenser pipette, such as Eppendorf's Multipette E3x, ensures adding the exact same volume to each well, and therefore makes imaging easier, as the same focal plane can be used for imaging all wells.

- Harvest transfected cells from:
  - 6-well plate
    - Remove media with a cell culture vacuum aspirator (or a pipette).
    - Add 5mL KK2-MC buffer with 2x pen/strep[31].
    - Remove buffer.
    - Add 500μL KK2-MC buffer with 2x pen/strep.
    - Harvest cells by gently pipetting up and down.
    - Collect cells in a 5mL round-bottom tube with a 35μm cell strainer snap cap.
  - SM5-agar plates
    - Using a 10μL loop, harvest the feeding front (vegetative cells) and transfer it to 1mL KK2-MC buffer with 2x pen/strep.
    - Spin down at 400xg, 2 min.
    - Resuspend in 500μL KK2-MC buffer with 2x pen/strep.
    - Collect cells in a 5mL round-bottom tube with a 35μm cell strainer snap cap.
- Sort individual cells onto the 24-well plate with SM5-charcoal-agar with *E. coli* B/r using the BD FACSAria Fusion Flow Cytometer sorter with BD FACSDiva software Version 8.0.1 (or another cell sorter).
  - For the FACSAria, we used the following settings:
    - FSC: 0V
    - SSC: 242V
    - 488-530_30: 362V
    - 561-610_20: 539V
    - 640-670_30: 466V
    - Nozzle: 100μL
    - Single cell sorting, target: 1 cell
    - Flow rate lower than 400 cells/s.
  - When possible, also sort positive and negative controls.
- Incubate plates at 22°C for 3 to 5 days.

6.2. Clone confirmation by imaging
- When generating fluorescent knock-ins or non-fluorescent knock-outs, image the 24-well plate using a fluorescent stereomicroscope[32].
- Positive clones can also be confirmed through PCR.

6.3. Clone confirmation by PCR
- Isolate genomic DNA from fruiting bodies:
  - Prepare a PCR strip, and add 20μL genomic DNA extraction buffer to each tube. Scale up reaction depending on the number of samples.

Reagent / solution	Volume
10x Taq buffer	1μL
IGEPAL	0.1μL
20mg/mL Proteinase K	0.05μL
Nuclease-free H_2O	18.85μL

  - Harvest 1-3 sori per well using a 10μL pipette tip.

[31] Antibiotics can be avoided if bacteria contamination of the sorter is not an issue.
[32] We recommend the Zeiss Axio Zoom.V16 fluorescent stereomicroscope.

- Dip the tip in the genomic DNA extraction buffer.
- Incubate at 56ºC for 45 min, and then at 95ºC for 10 min.
- gDNA extract can be stored at 4ºC up to 3 days, but best results are obtained when used immediately.

- PCR reaction:
  - Design primers flanking the targeted region. Aim for PCR products between 500 and 1000bp. For long inserts, two primer sets can be designed, spanning each junction. In the following example we use MR101/MR113 for the upstream junction, and MR112/MR103 for the downstream junction.

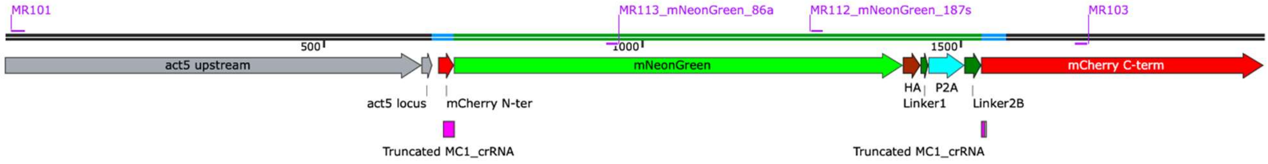

act5::mNeonGreen-P2A-mCherry

  - Use 2µL of genomic DNA from previous step to prepare the PCR reaction.

Reagent / solution	Volume
Taq 2X Master Mix	10µL
10µM Forward primers	0,4µL
10µM Reverse primers	0,4µL
Nuclease-Free H_2O	7,2µL
	18µL
Quick Genomic DNA extract	2µL

  - Perform confirmation PCR using the following settings:

Step	Temperature	Time
Initial Denaturation	95°C	2 min
Amplification: 35 cycles	95°C	30 sec
	T_a[33]	20 sec
	68°C	1 min
Final Extension	68°C	10 min
Hold	4–10°C	

  - Run 2µL of each PCR (with 2µL 6x loading dye and 6µL H_2O) on a 1% agarose gel to check PCR results. Below is an example of a confirmation PCR for 8 isolated clones of *D. discoideum* AX2 *act5*::*mNeonGreen-P2A-act5* (see also Appendix Figure S13), and the corresponding negative controls, with the primers MR101/113, targeting the upstream junction of the knock-in (Appendix Table S4).

[33] T_a might have to be adjusted depending on the primers and the amplified sequence. We successfully use Ta=56ºC for all the confirmation PCRs with the primer sets in Appendix Table S6.

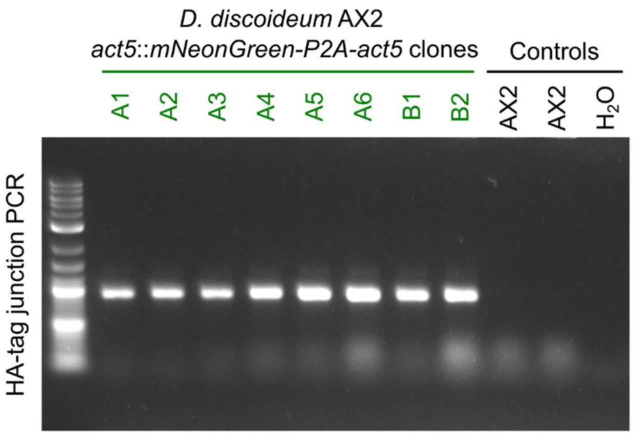

6.4. Sanger sequencing
- Clean the remaining PCR product (18µL) with a Qiagen QIAquick PCR purification column following manufacturer's instructions. Elute in 25µL EB and check purity with a NanoDrop Spectrophotometer.
- Adjust the concentration of the purified DNA following the recommendations of the sequencing company and send the samples for sequencing with one or both primers.

Step 7. Propagation of positive clones.

Clones confirmed by fluorescent microscopy and PCR can be stocked.

7.1. Propagate cells.
- Prepare regular SM5-agar plates.
- Spread 200µL of *E. coli* B/r in KK2 buffer at OD_{600}=2 and let it dry for 5 min.
- Pick one sorus from a positive well using a pipette tip.
- Spot this on the bacterial lawn.
- Incubate plates at 22°C for 3-7 days until the fruiting bodies cover the whole plate.

7.2. Optional: confirm purity of clone by flow cytometry analysis.
- Harvest some cells from the feeding front with a loop in 1mL KK2.
- Analyze by flow cytometry as detailed in Step 5.2[34].

7.3. Make glycerol stocks.
- Harvest sori using a 10µL loop
- Dip loop in 1mL of 25% glycerol/KK2-MC buffer.
- Store at -80°C.

[34] Expression of the fluorescent reporter will depend on the expression pattern of the targeted gene.

Part 2. Notes for CRISPR-Cas9 editing in other *Dictyostelid* species

Our CRISPR-Cas9 editing protocol can also be applied to other *Dictyostelid* species, but comes with several small adjustments. Below we specify these adjustments for each of the seven steps in our protocol.

Step 1. Identifying good CRISPR-Cas9 targets.

- Many *Dictyostelid* genomes are not available for popular tools like Cas-designer. When targeting a *Dictyostelid* genome different to *D. discoideum*, a custom tool for identifying CRISPR-Cas9 targets can be used. Alternatively, a conventional tool (e.g., Cas-designer) with the *D. discoideum* reference can be used. When using a conventional tool, search for potential off-target hits, for instance by Blasting your spacer and PAM sequence against the *Dictyostelid* genome of interest. Detecting all possible off-target hits for spacers with one or two nucleotide mismatches can be easily automated in Python.

Step 2. Designing donor oligos and donor PCR products.

- Same as for *D. discoideum.*

Step 3. Culturing *Dictyotelid*s.

- Most *Dictyostelid* species can be grown following the same protocol as described for non-axenic culturing of *D. discoideum*. Sometimes slightly different media conditions are recommended (e.g., LP-agar or SM5-agar with charcoal). Dictybase provides useful information there.

Step 4. Transfecting cells with RNP complex by electroporation.

- Cells were prepared and transfected following the same protocol as for non-axenic *D. discoideum*. Although we have not tested this, we can imagine that optimal electroporation conditions might sometimes differ between species. Growth rates on SM5-agar plates also differ between species, and therefore the appearance of a clear feeding front might take a different number of days: for example, for *T. lacteum*, it typically takes 5 to 7 days to get a clear feeding front, while for *D. purpureum*, it only takes 1 or 2 days and the plate is overgrown in 5 days. It is therefore important to account for such growth differences when transfecting multiple species in parallel.

Step 5. Flow cytometry analysis.

- The same parameters were used for all species. However, the gating slightly varied depending on the size/granularity of cells and the brightness of fluorescent reporters in the different species.

Step 6. Isolation of gene-edited cells using cell sorting.

- The same parameters were used for all species. However, the gating slightly varied depending on the size/granularity of cells and the brightness of fluorescent reporters in the different species.

Step 7. Propagation of positive clones.

- See Part 2, Step 3.

References

Bae S, Kweon J, Kim HS & Kim JS (2014a) Microhomology-based choice of Cas9 nuclease target sites. *Nat Methods* 11: 705–706

Bae S, Park J & Kim JS (2014b) Cas-OFFinder: a fast and versatile algorithm that searches for potential off-target sites of Cas9 RNA-guided endonucleases. *Bioinformatics* 30: 1473–1475

Baldauf SL, Romeralo M, Fiz-Palacios O & Heidari N (2018) A deep hidden diversity of *Dictyostelia*. *Protist* 169: 64–78

Concordet JP & Haeussler M (2018) CRISPOR: intuitive guide selection for CRISPR/Cas9 genome editing experiments and screens. *Nucleic Acids Res* 46: W242–W245

Hua Fu BX, Hansen LL, Artiles KL, Nonet ML & Fire AZ (2014) Landscape of target:guide homology effects on Cas9-mediated cleavage. *Nucleic Acids Res* 42: 13778–13787

Paix A, Folkmann A, Goldman DH, Kulaga H, Grzelak MJ, Rasoloson D, Paidemarry S, Green R, Reed RR & Seydoux G (2017) Precision genome editing using synthesis-dependent repair of Cas9-induced DNA breaks. *Proc Natl Acad Sci USA* 114: E10745–E10754

Paix A, Rasoloson D, Folkmann A & Seydoux G (2019) Rapid tagging of human proteins with fluorescent reporters by genome engineering using double-stranded DNA donors. *Current Protocols in Molecular Biology* 129: e102

Park J, Bae S & Kim JS (2015) Cas-Designer: a web-based tool for choice of CRISPR-Cas9 target sites. *Bioinformatics* 31: 4014–4016

Paschke P, Knecht DA, Silale A, Traynor D, Williams TD, Thomason PA, Insall RH, Chubb JR, Kay RR & Veltman DM (2018) Rapid and efficient genetic engineering of both wild type and axenic strains of *Dictyostelium discoideum*. *PLoS One* 13: e0196809

Sheikh S, Thulin M, Cavender JC, Escalante R, Kawakami SI, Lado C, Landolt JC, Nanjundiah V, Queller DC, Strassmann JE, *et al* (2018) A new classification of the Dictyostelids. *Protist* 169: 1–28

Zhu X, Ricci-Tam C, Hager ER & Sgro AE (2023) Self-cleaving peptides for expression of multiple genes in *Dictyostelium discoideum*. *PLoS One* 18: e0281211
